# Genome Sequence of *Lactobacillus pentosus* KCA1: Vaginal Isolate from a Healthy Premenopausal Woman

**DOI:** 10.1371/journal.pone.0059239

**Published:** 2013-03-19

**Authors:** Kingsley C. Anukam, Jean M. Macklaim, Gregory B. Gloor, Gregor Reid, Jos Boekhorst, Bernadet Renckens, Sacha A. F. T. van Hijum, Roland J. Siezen

**Affiliations:** 1 Department of Medical Laboratory Sciences, TWAS Research Unit, University of Benin, Benin City, Edo, Nigeria; 2 Canadian Research & Development Centre for Probiotics, Lawson Health Research Institute, London, Ontario, Canada; 3 Department of Biochemistry, University of Western Ontario, London, Ontario, Canada; 4 Departments of Microbiology and Immunology and Surgery, University of Western Ontario, London, Ontario, Canada; 5 Center for Molecular and Biomolecular Informatics, Radboud University Medical Centre, Nijmegen, The Netherlands; 6 TI Food and Nutrition, Wageningen, The Netherlands; 7 Netherlands Bioinformatics Centre, Nijmegen, The Netherlands; 8 Microbial Bioinformatics, Ede, The Netherlands; Columbia University, United States of America

## Abstract

The vaginal microbiota, in particular *Lactobacillus* species, play an important role in female health through modulation of immunity, countering pathogens and maintaining a pH below 4.7. We report the isolation and genome sequence of *Lactobacillus pentosus* strain KCA1 (formally known as *L. plantarum*) from the vagina of a healthy Nigerian woman. The genome was sequenced using Illumina GA II technology. The resulting 16,920,226 paired-end reads were assembled with the Velvet tool. Contigs were annotated using the RAST server, and manually curated. A comparative analysis with the available genomes of *L. pentosus* IG1 and *L. plantarum* WCFS1 showed that over 15% of the predicted functional activities are found only in this strain. The strain has a chromosome sequence of 3,418,159 bp with a G+C content of 46.4%, and is devoid of plasmids. Novel gene clusters or variants of known genes relative to the reference genomes were found. In particular, the strain has loci encoding additional putative mannose phosphotransferase systems. Clusters of genes include those for utilization of hydantoin, isopropylmalate, malonate, rhamnosides, and genes for assimilation of polyglycans, suggesting the metabolic versatility of *L. pentosus* KCA1. Loci encoding putative phage defense systems were also found including clustered regularly interspaced short palindromic repeats (CRISPRs), abortive infection (Abi) systems and toxin-antitoxin systems (TA). A putative cluster of genes for biosynthesis of a cyclic bacteriocin precursor, here designated as pentocin KCA1 (*penA*) were identified. These findings add crucial information for understanding the genomic and geographic diversity of vaginal lactobacilli.

## Introduction

Lactobacilli have long been known as an important constituent of a healthy vaginal ecology. Some differences may arise in species abundance among racial groups [1], [2]. For example, it has been shown that *L. iners* is often dominant in Caucasian and black African women [3]. Aberrations in the vaginal microbiota can result in bacterial vaginosis (BV), and higher rates of BV have been found in black women [4], [5], likely due to social and hygiene practices [6]–[8]. We isolated a strain of *Lactobacillus pentosus* and designated it KCA1. Like a number of other vaginal *Lactobacillus* strains developed as probiotics, KCA1 was shown to produce biosurfactants, hydrogen peroxide (H_2_O_2_), and inhibit the growth of intestinal and urogenital pathogens [9], as well as exhibit varying degrees of acid and bile tolerance [10].

Initially, on the basis of a carbohydrate-fermentation test and information from 16S rRNA gene sequencing, this bacterium was identified as *L. plantarum*. However, following the recommendation of Bringel et al. [11] we reclassified the isolate as *L. pentosus* KCA1 on the basis of the gene sequences of *recA* (recombinase A), *dnaK* (heat shock protein HSP70) and *pheS* (phenylalanyl-tRNA synthase alpha subunit), as these genes have the most discriminatory power in distinguishing the species and subspecies of *L. plantarum* and *L. pentosus* [12], [13].

*Lactobacillus pentosus* is a versatile species found in a variety of environmental niches, including dairy, meat, and vegetable/plant ferments. For example *L. pentosus* strain b240 originally isolated from fermented tea leaves [14], has been shown to have immuno-modulatory probiotic potential [15]. African diets contain many different types of lactic acid bacteria in fermented foods [16].

Recently, the draft genome sequences of *L. pentosus* MP-10 [17] and *L. pentosus* IG1 [18] have been published, while the genome of *L. plantarum* WCFS1 has been re-sequenced and re-annotated [19]. These data provide important information that has allowed us to describe the first genome sequence and annotation of an African vaginal isolate, *Lactobacillus pentosus* KCA1.

## Results and Discussion

### General Genome Features

**Figure 1:**
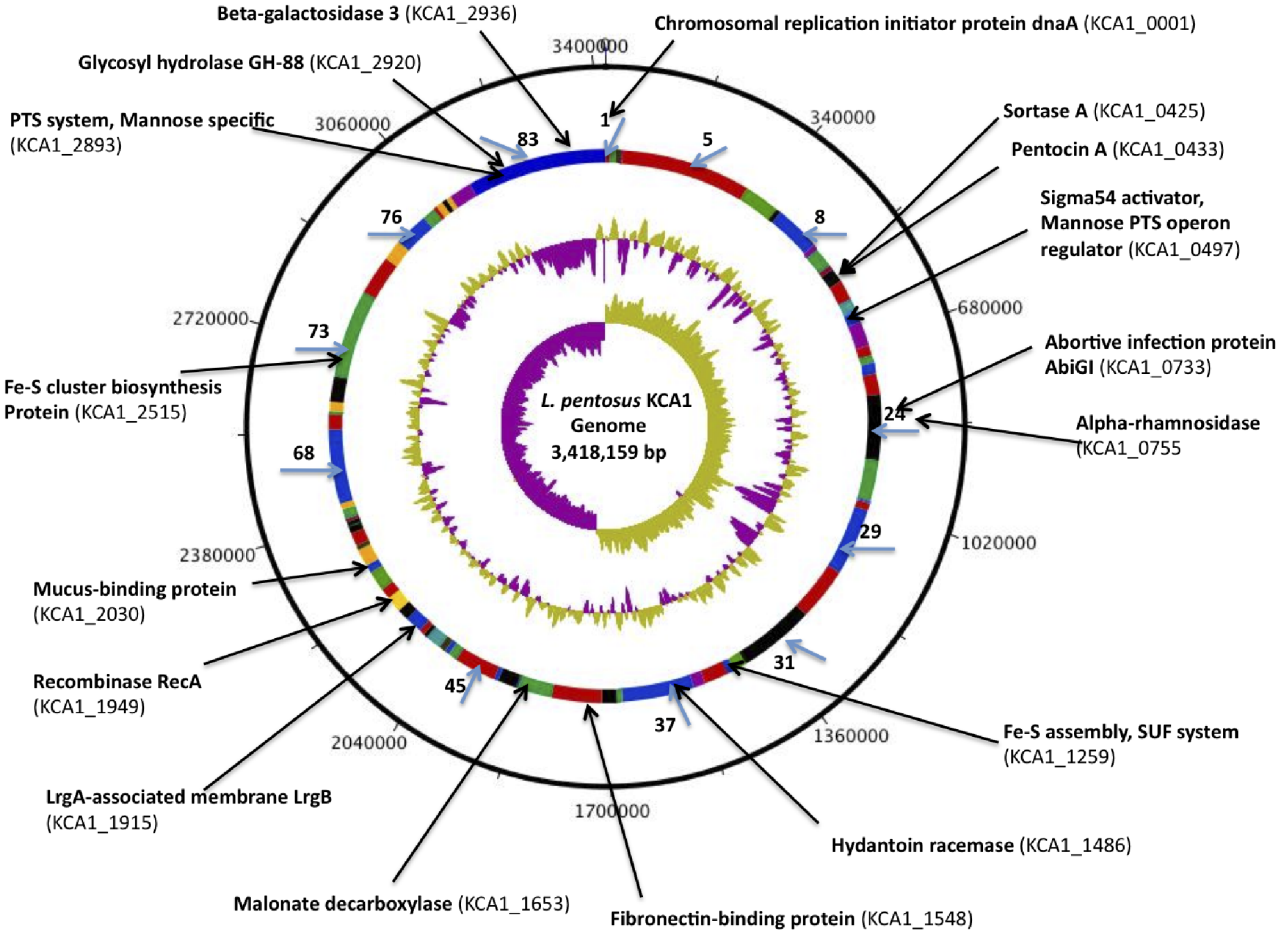
Genome atlas view of the scaffolded contigs of *L. pentosus* KCA1. From the outer circle inward: The first ring shows the entire chromosome. The second ring shows the location of the 83 contigs based on *L. plantarum* WCFS1 genome order/orientation as template. The black arrow-heads indicates the position of some of the genes of interest located in the corresponding contigs described in the text with the locus tag in bracket. The fourth ring shows the local %GC plot and the innermost circle shows the GC-skew with sharp changes occurring at the origin and terminus of replication. The Atlas was constructed using DNA plotter.

**Figure 2:**
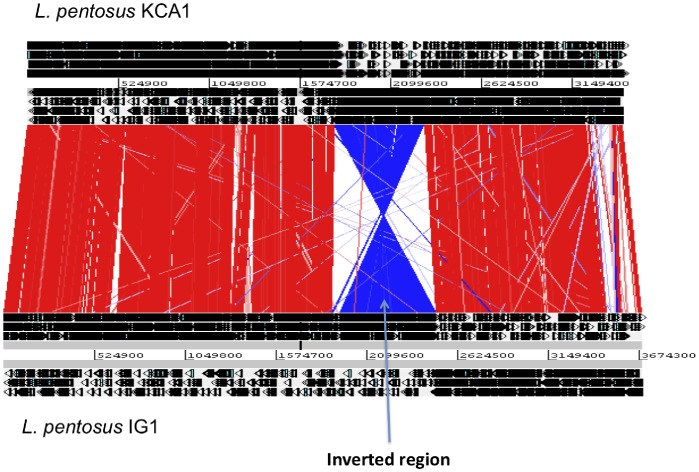
Whole genome alignment of *L. pentosus* KCA1 with *L. pentosus* IG1. Constructed with the ACT tool. Red lines indicate orthologous genes in the same orientation. Blue lines indicate orthologous genes in reverse orientation. The large inverted region in *L. pentosus* IG1 is indicated.

The draft genome sequence of *Lactobacillus pentosus* KCA1 consists of 3,418,159 nucleotide base pairs in 83 contigs. No contigs were present at greater than expected coverages, suggesting that this strain is devoid of plasmids. The genome features are presented in **Table 1** and **Figure 1**. The order of genes (synteny) is similar to *L. pentosus* IG1 and to *L. plantarum* WCFS1, despite the variable and lower nucleotide sequence identity observed in the three housekeeping genes in Table 2. While there are only a few regions where rearrangements occur relative to *L. plantarum* WCFS1, there appears to be a large inversion in the published *L. pentosus* IG1 genome, as shown in **Figure 2**. However, this does not affect comparisons of open reading frames (ORFs).

**Table 1:** Comparison of sequenced genomes of L. pentosus, L. plantarum and L. iners strains.

Strain	*L. pentosus* KCA1	*L. pentosus* MP-10	*L. pentosus* IG1	*L. plantarum* WCFS1	*L. plantarum* JDM1	*L. plantarum* ST-III	*L. plantarum* ATCC 14917	*L. iners* AB-1
**Chromosome size (bp)**	**3,418,159**	3,835,873	3,982,537	3,308,273	3,197,759	3,254,376	3,198,760	1,304,000
**GC content (%)**	**46.4**	46	44.9	44.5	44.6	44.5	44.5	32.7
**CDS (protein coding sequence**	**2967**	2,755	2765	3,042	2,948	3,013	3,154	1,190
**rRNA**	**5**	3	1	5	5	5	5	6
**Plasmids**	**0**	3	7	3	2	1	0	0
**No. Contigs**	**83**	90	13 (Scaffolds)	1	1	1	36	7 scaffolds
**Status of assembly**	Draft	Draft	Draft	Finished	Finished	Finished	Draft	Draft
**Source**	Human vagina	Alorena green table olive	Spanish-style greenolive	Human saliva	Chinese probiotic	Chinese probiotic Kimchi	Human GUT	Human vagina

**Table 2:** Summary of housekeeping genes sequence identity matrices (%) to pentosus and plantarum strains.

*L. pentosus* KCA1	*L. plantarum* WCFS1	*L. plantarum ssp plantarum* LMG 6907	*L. plantarum ssp argentoratensis* LMG 9205	*L. pentosus* IG1	*L. paraplantarum* LMG 16673	*L. fabifermentans* DSM 21115
***recA*** ** (KCA1_1949)**	92	87	87	**97**	86	**ND**
***dnaK*** ** (KCA1_1717)**	90	90	90	**96**	90	87
***pheS*** ** (KCA1_1326)**	84	83	83	**93**	84	ND

ND = Not Done.

All predicted genes, proteins, enzymes and their functions are putative as are pseudogenes. The *L. pentosus* KCA1 genome is predicted to contain 2992 protein-encoding ORFs, of which 25 are putative pseudogenes representing fragments of proteins, leaving 2967 as putative protein-coding genes that appear in the NCBI non-redundant database. This exceeds previous comparative genomic studies that estimated the number of predicted protein-coding genes in lactic acid bacteria (LAB) to be from 1,700 to over 2,800 [20]. This difference suggests a large amount of gene gain in the *L. pentosus* KCA1 lineage. In comparison, *L. iners* AB-1 genome, a vaginal isolate, appears to have undergone a large genome reduction phase, as it has only 1190 predicted ORFs [21]. The G+C content of the *L. pentosus* KCA1 genome is 46.4%, which is slightly higher than *L. pentosus* IG1 (44.6%), *L. pentosus* MP-10 (46.0%) and *L. plantarum* strains [*L. plantarum* WCSF1 (44.5%), *L. plantarum* JDM1 (44.6%), *L. plantarum* ST-III (44.5%), *L. plantarum* ATCC 14917 (44.5%), and *L. iners* AB-1 (32.7%)], as shown in **Table 1**.

Functional classification of the predicted genes by Clusters of Orthologous Groups (COGs) of genes [22] showed that 2349 (79.1%) were homologous to known gene families, including 300 (10.1%) identified as ‘general function predictions only’ and 216 (7.3%) poorly characterized gene functions designated as “functions unknown”, while 817 (27.5%) do not have any COG association (**Figure S1**). The *L. pentosus* KCA1 genome contains 5 rRNA operons, which is the same as sequenced *L. plantarum* strains. The genome encodes 52 putative ribosomal proteins as shown in **Table S1**. Comparatively, in *L. pentosus* IG1 there is only a single predicted copy of the 16S and 23S rRNAs, three copies of the 5S rRNA, and 44 predicted tRNAs [18], unlike *L. iners* AB-1 which has six rRNA gene operons [21].

### Phylogenetic Relationships to other *L. plantarum* and *L. pentosus* Strains

**Figure 3:**
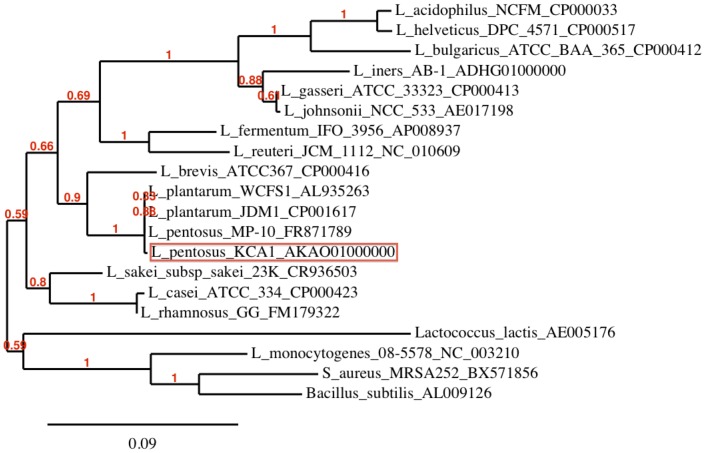
Phylogenetic tree of *Lactobacillus* species showing the position of *L. pentosus* KCA1 based on the 16S rRNA gene sequences. The numbers at the end of each strain indicates the accession number. Sequences were aligned with MUSCLE [58], and unreliable positions were curated using Gblocks [59]. A maximum likelihood tree was generated by PhyML using the GTR substitution model [60] and allowing 4 rate substitution categories. Confidence values for the branching order were generated by bootstrapping (based on 100 replications). The number at the nodes indicates the bootstrap values. The scale bar indicates 1 nucleotide substitution per 100 nucleotides.

**Figure 4:**
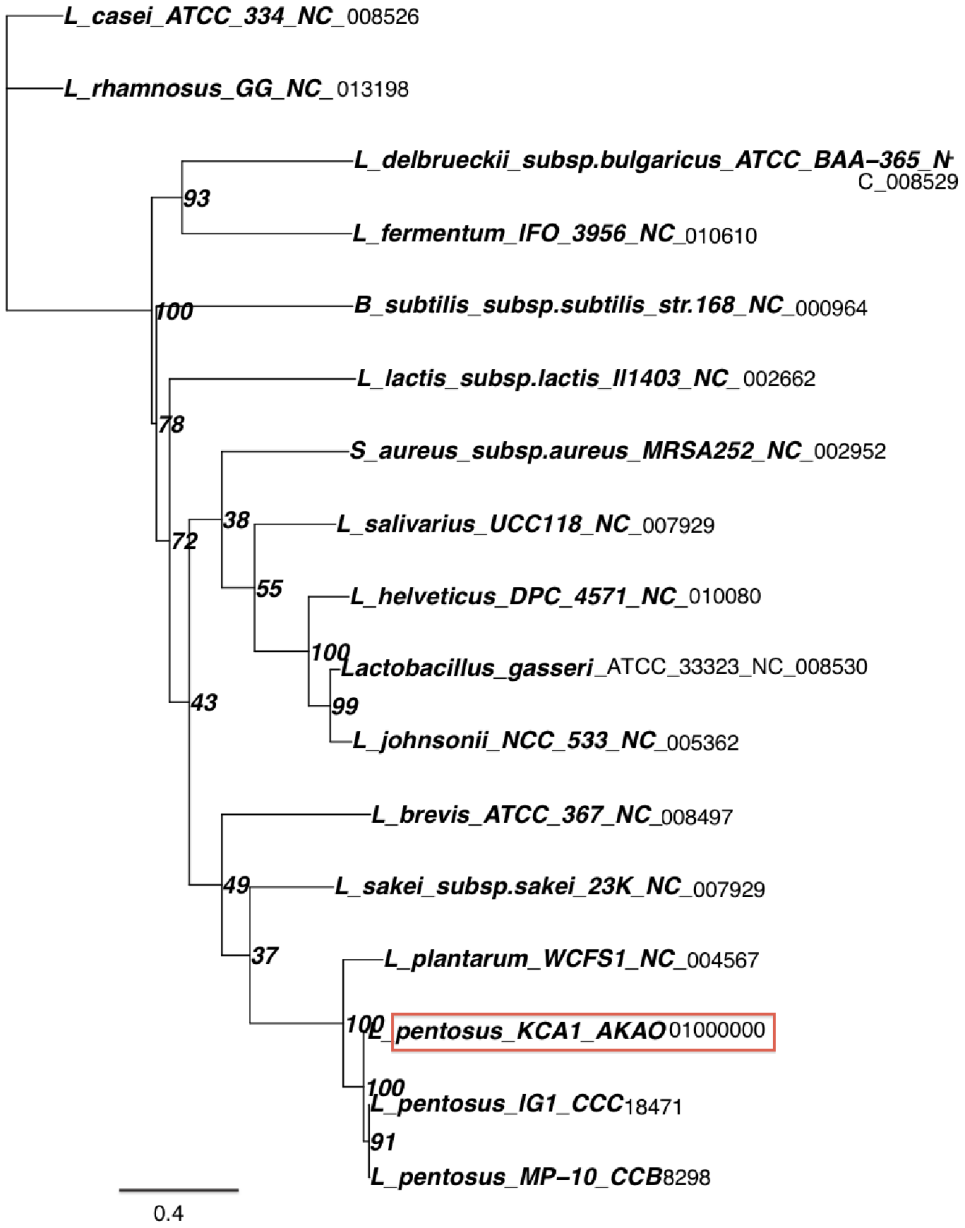
Phylogenetic tree of Lactobacillus species showing the position of *L. pentosus* KCA1 based on the sequences of the housekeeping gene recA, relative to other plantarum and pentosus strains. The numbers at the end of each strain indicates the accession number. Sequences were aligned with MUSCLE [58], and unreliable positions were curated using Gblocks [59]. A maximum likelihood tree was generated by PhyML using the GTR substitution model [60] and allowing 4 rate substitution categories. Confidence values (%) for the branching order were generated by bootstrapping (based on 100 replications). The number at the nodes indicated the bootstrap values. The scale bar indicates 1 nucloetide substitution per 100 nucleotides.

The phylogenetic position of *L. pentosus* KCA1 was determined from its 16S rRNA gene sequence, relative to other selected 16S rRNA gene sequences obtained from the National Center for Biotechnology Information (NCBI) database (**Figure 3**). The phylogenetic tree shows that *L. pentosus* KCA1 cannot be distinguished from *L. pentosus* and *L. plantarum* strains based on 16S rRNA sequence, as the relationship of the four branches at the node identifying this clade is unresolved. However, gene trees of the three conserved (housekeeping) genes (*recA, dnaK, pheS*) suggests that *L. pentosus* KCA1 is closer to *L. pentosus* IG1 and *L. pentosus* MP-10 with higher percentage identity than to *L. plantarum* WCFS1 (**Figure 4**, **Table 2** **and Table S2**). Although, identity value is lower than would be expected if it belongs to the same subspecies, it will probably not be feasible to define a new subspecies for pentosus just based on one strain, as the identity to pentosus is not over the 98% level for these genes. These housekeeping genes have been shown to have the most discriminatory power in distinguishing the species and subspecies of *L. pentosus* and *L. plantarum* respectively [11]–[13].

### Unique Carbohydrate Metabolism

*L. pentosus* KCA1 encodes 457 putative genes for carbohydrate metabolism, which is consistent with the taxonomy as a heterofermentative lactic acid bacterium. Among these, 19 gene cassettes for carbohydrate utilization can be distinguished in a 115 kb region or “sugar island”, based on gene content, operon structure, and BLASTp hits [23]. This region encodes 91 putative proteins that are unique to *L. pentosus* KCA1 relative to *L. pentosus* IG1, *L. pentosus* MP-10 and *L. plantarum* WCFS1.

We identified phosphotransferase systems (PTS) for many different sugars, e.g. fructose, glucose/sucrose, trehalose, cellobiose, beta-glucosides, mannose, and a novel locus coding for a putative glycerol-3-phosphate ABC transporter (KCA1_1144-KCA1_1147). The genome has many ORFs that appear to be involved in mannose metabolism, including an ORF that likely encodes a putative regulator ManR of the mannose operon, a mannose-6-phosphate isomerase, and three mannose-specific PTS systems (KCA1_0493–0499, KCA1_2893–2896, KCA1_2935–2940). This is supported by the presence of two extra putative gene clusters coding for mannose/fructose/sorbose specific PTS system EIIA-EIID components (KCA1_2870–2873, KCA1_2961–2964); some of these mannose PTS systems are unique to *L. pentosus* KCA1, and are not in *L. pentosus* IG1 or *L. plantarum* WCFS1. The *L. pentosus* KCA1 genome encodes several novel putative gene cassettes for carbohydrate utilization (**Table S3**). One encodes α-L-rhamnosidase, β-glucosidase, glycoside hydrolase family 43, a regulator, and a MFS family transporter (KCA1_2348-KCA1_2345), including a novel tannase (tannin acylhydrolase) (KCA1_2422). In the sugar island, seven novel putative genes were predicted to code for different unsaturated glucuronyl hydrolases of the glycosyl hydrolase families GH-88 and GH-28.

### Horizontal Gene Transfer (HGT)

We identified potentially foreign genes as ORFs with a best BLASTn hit to the NCBI non-redundant database that was not in the *Lactobacillus* genus. KCA1 has 180 predicted genes possibly acquired from organisms outside its genus, accounting for 6% of the protein-coding sequences, compared to 65 genes identified as HGT in *L. iners* AB-1 accounting for 5.5% [21]. Of the 180 predicted foreign genes in *L. pentosus* KCA1, 18 (10%) have at least 70% amino acid identity to a non-*Lactobacillus* organism including six to *Enterococcus faecium*, five *Pediococcus acidilacti*, three *Streptococcus gallolyticus* and one *Oenococcus Oeni* ATCC BAA-1163, *Mitsuokella multacida* DSM 20544, and *Listeria monocytogenes* str. 1/2a F6854. Although a few of the most similar alleles of some *L. pentosus* KCA1 genes are found in *Enterococcus, Pediococcus, Streptococcus* and *Oenococcus* species, these genera are closely related. This pattern of similarity could be due to other evolutionary processes such as duplication, differential loss, or differing evolutionary rates.

In terms of COG distribution of the putative HGT genes, 30 (16.7%) belong to COG class G responsible for carbohydrate transport and metabolism, while 45 (25%) had no COG class. Several of the horizontally acquired genes are unique to *L. pentosus* KCA1 relative to *L. pentosus* IG1 and *L. plantarum* WCFS1. For example, the genome has a novel five gene cluster encoding putative hydantoin racemase (KCA1_1486) with 42% amino acid identity to *Thermoanaerobacter brockii* subsp. *finnii* Ako-1, and a N-methylhydantoinase (KCA1_1489)-(ATP-hydrolyzing) with 61% amino acid identity to *Enterococcus faecalis* E1Soi. It appears that hydantoin racemase is present only in the genome of *L. pentosus* KCA1 among all the known *Lactobacillus* bacteria, as shown in **Figure S2**. The gene is located within a cassette involving an ATP-hydrolyzing N-methylhydantoinase and a putative protein involved in hydantoin/pyrimidine utilization.

These findings are interesting because species such as Listeria and Thermoanaerobacter can be found in food and feces, but not in the vagina. This suggests potential interaction of KCA1 with food or gut organisms prior to vaginal colonization.

### Polyglycan Utilization

Novel putative genes were found for polyglycan utilization belonging to the glycosyl hydrolase (GH) families GH-28 (KCA1_2999), GH-88 (KCA1_2923, KCA1_2920), and GH-43 (KCA1_2347). The known activities of the GH-28 family encompasses the predicted genes in KCA1 for polygalacturonase (KCA1_2904; KCA1_2900) and rhamnogalacturonase (KCA1_2888; KCA1_2907). Two genes (KCA1_2923 and KCA1_2920) belong to glycosyl hydrolase family GH-88; KCA1_2923 is 378 amino acids long and has a best hit (46% amino acid identity) to *Paenibacillus sp.* JDR-2, while KCA1_2920 is 368 aa long and has 55% amino acid identity to *Enterococcus faecium* DO. Comparatively, *L. iners* AB-1, a vaginal isolate has a gene that belongs to glycosyl hydrolase family 31 [21]. These hydrolase genes in *L. pentosus* KCA1 suggest the strain may have adapted to the mucin turn-over of the vaginal mucosa, which is primarily made of mucin glycoproteins containing monosaccharide chains of L-fructose, N-acetylneuraminic acid (sialic acid), galactose, N-acetyl-galactosamine, and N-acetylglucosamine [24]. The *L. pentosus* KCA1 encodes a putative gene cassette for glycogen metabolism which includes a GH-13-type 1,4-alpha-glucan (glycogen) branching enzyme GlgB (KCA1_0017), glucose-1-phosphate adenylyltransferase regulatory subunit GlgD (KCA1_0019), enzymatic subunit GlgC (KCA1_0018), glycogen synthase, ADP-glucose transglucosylase GlgA (KCA1_0020), glycogen phosphorylase GlgP (KCA1_0021), and maltodextrin glucosidase MalZ (KCA1_0022). The vaginal epithelium is covered with large amounts of glycogen, which is induced by estrogen during premenopausal period. This may indicate good adaptation of KCA1 to the vaginal environment.

### Malonate Decarboxylation

*L. pentosus* KCA1 encodes all the putative enzymes for decarboxylation of malonate to acetate. Malonate is a three-carbon dicarboxylic acid and a competitive inhibitor of succinate dehydrogenase [25]. The gene cassette contains membrane-integrated, biotin-dependent, energy-conserving Na^+^ translocating enzymes with an integral membrane protein (KCA1_1656), regulated by a LysR*-*family transcriptional regulator, (*mdcR* KCA1_1655) as shown in **Figure S3**. This is followed by the malonate decarboxylase subunits including the epsilon (*mdcH*), alpha (*mdcA*), delta (*mdcC*), beta (*mdcD*), and gamma (*mdcE*) subunits (KCA1_1654–1651) having 92–99% identity to *L. pentosus* MP-10 and *L. pentosus* IG1. It is feasible that lactobacilli encounter malonate in the gut in people consuming legumes, but it remains to be determined if malonate is present in the vagina.

### Phage Defense Systems

As a bacterial immune system against foreign DNA, CRISPRs evolve rapidly in response to changing phage pools [26]. Two CRISPR-associated sequence (Cas) systems were identified in *L. pentosus* KCA1, possibly reflecting exposure to phage in the vagina [27]. CRISPR systems are present in the *L. pentosus* MP-10 and *L. pentosus* IG1 genomes but not found so far in any of the sequenced *L. plantarum* strains. *L. iners* AB-1 and vaginal *L. johnsonii* and *L. gasseri* lack CRISPR regions and the associated cas genes [21]. CRISPR1 and CRISPR2 consist of 4 and 8 *cas* genes respectively. Cas1 and Cas2 genes are absent in *L. pentosus* IG1, but similar sets of the 8 *cas* genes are found in *L. crispatus* ST1, *L. casei* ATCC 334, *L. delbrueckii subsp. bulgaricus* ATCC 11842 and *L. fermentum* IFO 3956.

There is another phage resistance property, accomplished through an abortive infection (Abi) system, that can target different phases of phage development [28]. At least three complete AbiGI-AbiGII systems are predicted in *L. pentosus* KCA1 (**Table 3**), but they appear to be incomplete in *L. pentosus* IG1 and absent in other sequenced *L. plantarum* strains and *L. iners* AB-1.

**Table 3:** Phage resistance via abortive infection proteins predicted in L. pentosus KCA1.

KCA1 gene	Size (AA)	*L. pentosus* KCA1 product	Best BLASTp hit
KCA1_0733	197	Abortive infection protein AbiGI	(97% id) to *L. pentosus* IG1
KCA1_0734	52	hypothetical protein	No NCBI BLASTp hit
KCA1_0735	231	Abortive infection protein AbiGII	(40% id) to *L. crispatus*(absent in *L. pentosus* IG1)

KCA1_0859	248	Abortive infection protein AbiGII (putative)	(38% id) to *L. rhamnosus*(absent in *L. pentosus* IG1)
KCA1_0860	197	Abortive infection protein AbiGI	(46% id) to *L. rhamnosus*

KCA1_2390	433	Abortive infection protein, ATPase	(97% id) to *L. pentosus* IG1

KCA1_2801	229	Abortive infection protein AbiGI	(38% id) to *L. brevis*(absent in *L. pentosus* IG1)
KCA1_2802	281	Abortive infection protein AbiGII	(49% id) to *L. brevis*(absent in *L. pentosus* IG1)

### Toxin-antitoxin System

Toxin-antitoxin (TA) systems are widely distributed in prokaryotes, and some often have them in multiple copies [29]. There are seven complete putative TA systems in *L. pentosus* KCA1. In comparison, *L. plantarum* WCFS1 contains only one complete TA system [19]. They belong to distinct families (**Table 4**). Chromosomal homologs of these TA systems have been found to induce reversible cell cycle arrest or programmed cell death in response to starvation or other adverse conditions [30]. The genes KCA1_0730–731 encode a putative TA system of the xre/HigA/VapI-HigB family, which has been shown to be involved in stress responses to antibiotics, especially chloramphenicol [31] and kanamycin [32], to which *L. pentosus* KCA1 are resistant [9]. These antibiotics are widely used in Nigeria. This TA phenotype contributes to the tolerance of biofilm bacteria to antibiotics [33]. Other toxin genes are found to be highly induced in persister cells, including RelE, (KCA1_0922), HigB (KCA1_0730), MazF (KCA1_0440), and YoeB (KCA1_2899) [34].

**Table 4:** Toxin-antitoxin systems predicted in L. pentosus KCA1.

*L. pentosus* KCA1 gene	Amino Acid Length	Annotation product in *L. pentosus* KCA1	*L. pentosus IG1*
KCA1_0363	70	toxin-antitoxin system, toxin, *HigB* family	Absent
KCA1_0364	97	toxin-antitoxin system, antitoxin, *HigA* family	Present
KCA1_0440	130	toxin-antitoxin system, toxin component, DNA-binding protein, *PemK/MazF* family	Present
KCA1_0258	95	toxin-antitoxin sytem, antitoxin, *RelB/DinJ* family	Present
KCA1_0862	78	toxin-antitoxin system, antitoxin component, *phd* family	Present
KCA1_0863	223	toxin-antitoxin system, toxin component, zeta toxin family	Present
KCA1_0922	109	toxin-antitoxin system, toxin component, *RelE* family (Plasmid stabilization system protein)	Absent
KCA1_0923	105	toxin-antitoxin system, antitoxin component, *RelB* family (Plasmid stabilization system protein)	Present
KCA1_2816	133	toxin-antitoxin system, toxin component, *Fic/Doc* family	Present
KCA1_2817	82	toxin-antitoxin system, antitoxin component, *AbrB* family	Present
KCA1_2898	92	toxin-antitoxin system, antitoxin, *RelB/DinJ* family	Absent
KCA1_2899	85	toxin-antitoxin system, toxin, *Txe/YoeB* family	Absent
KCA1_0730	107	toxin-antitoxin system, toxin component, *HigB* family	Absent
KCA1_0731	106	toxin-antitoxin system, antitoxin, *xre/HigA/VapI* family	Present

### LytSR-LrgAB System

The *L. pentosus* KCA1 genome harbors a putative LytSR two-component regulatory system found in *L. pentosus* IG1 and *L. pentosus* MP-10, but not in sequenced *L. plantarum* strains. The LytSR may help *L. pentosus* KCA1 develop a biofilm or integrate into a multi-species one. The operon contains the autolysis histidine kinase LytS (KCA1_1912), autolysis response regulater LytR (KCA1_1913), antiholin-like protein LrgA (KCA1_1914), and LrgA-associated membrane protein LrgB (KCA1_1915). Importantly, these operons play roles in biofilm development by controlling the release of genomic DNA, an important structural component of the biofilm matrix [35]. The ability of lactobacilli to penetrate and disrupt BV biofilms could be important in maintenance of a healthy vagina [36].

### Bacteriocin

**Figure 5:**
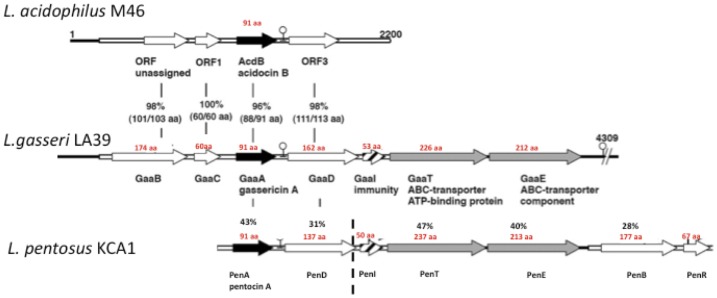
Class V bacteriocin biosynthesis gene cassettes and their organization in three *Lactobacillus* species including *L. pentosus* KCA1. PenA represents the pentocin KCA1 precursor. Adapted from Kawai et al, 2009, AEM 75∶1324–1330.

The genome of *L. pentosus* KCA1 contains a 7-gene cluster for biosynthesis of a putative class V cyclic bacteriocin precursor, here designated as pentocin KCA1 *penA* (KCA1_0433, **Figure 5**). The bacteriocin shows 49% amino acid (aa) residue identity to the circular class IIc bacteriocin gassericin A from *L. gasseri* LA39 [37], 50% aa identity to acidocin B from *L. acidophilus* [38], 34% aa identity to butyrivibriocin AR10 from *Butyrivibrio fibrisolvens* [39], and 38% aa identity to an unknown bacteriocin of *Streptococcus sp*. 2_1_36FAA [40]. The locus also encodes two hypothetical proteins (PenD and PenB), a PBSX family transcriptional regulator (PenR), and an accessory ABC transporter (PenE and PenT). The presence of an entire synthetic and secretory gene cluster suggests an important role for this product. A number of bacteriocins have been reported in vaginal bacteria, but the extent to which they influence the microbiota composition remains to be determined.

### Cell-surface Proteins (Secretome)

*Lactobacillus* cell-surface proteins can aid in governing interactions with the host and bacterial environments [41]. KCA1 has a variety of (about 15–20) novel putative cell-surface proteins relative to *L. plantarum* WCFS1.

The LAB-Secretome database, http://www.cmbi.ru.nl/lab_secretome was used to predict the secretome of *L. pentosus* KCA1, including all the predicted extracellular proteins and their La(Lactobacillales)COG classification. Of the 2967 proteins predicted in *L. pentosus* KCA1, 276 (9.3%) were predicted to belong to the secretome [42], of which 31 (11.3%) are LPXTG cell-wall anchored. The encoded sortase A enzyme (KCA1_0425) that is LPXTG specific has 100% amino acid identity to sortases of *L. pentosus* IG1 and *L. pentosus* MP-10. Sixty two proteins (22.3%) are predicted to be lipid-anchored, 125 proteins (45.5%) N-terminally anchored, 42 (15.2%) secreted/released, and 5 (1.8%) C-terminally anchored. Of the 276 cell surface proteins, 262 (95%) are associated with several LaCOG families (data not shown).

Examination of the *L. pentosus* KCA1 genome showed putative adhesion factors and conserved adhesion domains commonly found in lactobacilli. Mucus-binding proteins are common to lactobacilli that colonize the gastro-intestinal tract [43]. A novel putative mucus-binding protein with LPXTG cell-wall anchored motif (KCA1_2505) is 517 amino acids long, and was found to be a member of LaCOG01470. This truncated mucus-binding protein has 42% amino acid identity to adherence-associated protein (AapA) found in *L. plantarum* WCFS1 having 1356 amino acids. KCA1_1405 has 56% amino acid identity to a mucus-binding protein of *L. plantarum* WCFS1, lp_1643 (2219 amino acids) [44] and 30% amino acid identity to a mucus-binding protein of *L. reuteri* (3269 amino acids) reported to specifically bind mucus glycoproteins [45]. An interesting feature of mucus-binding protein KCA1_1405 is that it is 2295 amino acids long, the largest open reading frame in the *L. pentosus* KCA1 genome. A putative fibronectin/fibrinogen-binding protein FbpA (KCA1_1548) was found, which may also be involved in adherence [46].

### Exopolysaccharide Biosynthesis

The *L. pentosus* KCA1 genome encodes several gene clusters for exopolysaccharide biosynthesis, encoding putative enzymes belonging to the glycosyltransferase family 2, which may be horizontally acquired due to the presence of mobile insertion sequences (KCA1_0963–KCA1_0965 transposase IS3 family) (**Table S4**). There appears to be a diversity in EPS gene cassettes [23] indicating that lactic acid bacteria contain a vast pool of glycosyltransferases with a wide range of sugar and linkage specificities. Notably, some EPS/CPS (capsular polysaccharides) genes of *L. pentosus* KCA1 are not the same as in *L. plantarum* WCFS1 or *L. plantarum* JDM1 as described above and shown in **Figure S4**. *L. plantarum* WCFS1 has 3 consecutive EPS/CPS gene clusters, separated by transposases (T); gene cluster cps3 is present in *L. pentosus* KCA1, and part of cps2 is also present in *L. pentosus* KCA1. A similar variability of EPS gene cassettes has been observed in other LAB [47] presumably leading to variation in the structure of capsular and exopolysaccharides. Previous studies have demonstrated the adherence of *Lactobacillus* species producing exopolysaccharides to vaginal cells [48].

### Amino Acid Biosynthesis and Biodegradation

*L. pentosus* KCA1 contains predicted genes that encode the biosynthetic pathways required for the synthesis of the majority of the amino acids *de novo*. For example: serine from pyruvate by using L-serine dehydratase, (KCA1_0417–418) and D-serine dehydratase (KCA1_2271), which has 100% amino acid identity to an ortholog in *L. pentosus* IG1. Several putative enzymes are encoded for interconversion of L-aspartate and L-aspargine: two putative genes code for asparagine synthetase AsnB [glutamine-hydrolyzing] (KCA1_0784, KCA1_2527) and also two genes for aspartate-ammonia ligase AsnA (KCA1_0765, KCA1_2313). In addition, there are 13 putative genes dedicated to glutamate metabolism, e.g. a glutamine synthetase type I GlnA (KCA1_1348) that can convert L-glutamate to L-glutamine in the presence of ammonia. The pathways for the biosynthesis of the branched-chain amino acids, isoleucine, leucine, and valine were reported to be clearly absent in *L. plantarum* WCFS1 [44]. However, *L. pentosus* KCA1, similar to *L. pentosus* IG1, has the five genes for complete biosynthesis of L-leucine from pyruvate metabolism. The ORFs were annotated as *2-*isopropylmalate synthase LeuA (KCA1_1493], 3-isopropylmalate dehydrogenase LeuB (KCA1_1494), 3-isopropylmalate dehydratase large subunit LeuC (KCA1_1495) 3-isopropylmalate dehydratase small subunit LeuD (KCA1_1496) and branched-chain amino acid aminotransferase BcaT (KCA1_2018). The primary enzyme required for protein and polypeptide utilization, the extracellular protease Prt that is involved in primary breakdown of proteins, is lacking in the *L. pentosus* KCA1 genome similar to *L. plantarum* WCFS1 [44]. *L. pentosus* KCA1 has 12 putative genes encoding intracellular peptidases of different specificity, including five dipeptidases.

### Stress Tolerance

*L. pentosus* KCA1 has the capacity to survive adverse conditions associated with the human vagina such as low pH, as shown *in vitro* [10], similar to *L. iners* AB-1 [21]. In support of this, *L. pentosus* KCA1 encodes eight putative genes for Na+/H+ antiporters which could be involved in acid stress response as in *L. pentosus* IG1 [18] and *L. plantarum* WCFS1 [44]. The gene cluster involving eight putative genes (KCA1_1998–KCA1_2005) codes for H (+)-transporting two-sector ATPases, which may serve as a major regulator of intracellular pH.

The genome encodes two putative alkaline shock proteins Asp1 and Asp2 (KCA1_0751, KCA1_0750), similar to *L. iners* AB-1 [21] and a general stress protein, Gls24 family (KCA1_1363) that may play a role in pH homeostasis. KCA1 has 16 putative genes in the heat-shock operon, encoding a heat-inducible transcriptional repressor HrcA (KCA1_1719) and molecular protein chaperones GrpE (KCA1_1718), DnaK (KCA1_1717), and DnaJ (KCA1_1716). In addition to the GroEL (KCA1_0569)-GroES (KCA1_0568) chaperonin encoding the heat shock proteins of the Hsp60 family, *L. pentosus* KCA1 encodes three small heat shock proteins and chaperonin Hsp33 (heat shock protein 33) (KCA1_0469) plus a novel S4-domain-containing ribosome-associated heat shock protein (KCA1_0460), and heat shock protein HtpX (KCA1_0427) which is a cell-surface zinc metalloproteinase. KCA1 also encodes four putative cold shock proteins including 2 CspA (KCA1_0934, KCA1_0027), CspC (LPKCA1_1293) and a cold-shock DEAD-box-protein, which is associated with an ATP-dependent RNA helicase (KCA1_0430). Nine putative universal stress proteins of the UspA family and two putative stress-responsive transcription regulators (KCA1_0121, KCA1_0589) were identified.

### Regulation

*L. pentosus* KCA1 encodes 236 putative regulatory genes (∼8% of the total proteins) some of which are involved in stress response. The genome contains DNA-directed RNA polymerase, sigma factor 30 SigH (KCA1_0522) and sigma-54 factor, transcriptional regulator containing an AAA-type ATPase domain (KCA1_2897) which is absent in *L. pentosus* IG1 but has a best BLASTp hit (57% identity) to SipR of *Lactobacillus casei* BL23 that directs the enzyme to a specific promoter. The sigma factor 30 SigH is 187aa long and it has only 60.9% identity to DNA-directed RNA polymerase, sigma-H factor of *L. plantarum* WCFS1. Other sigma factors, active under different stress conditions, regulate the transcription of various stress response genes such as the RNA polymerase sigma factor 42 RpoD (KCA1_1678) in addition to RNA polymerase sigma-54 factor RpoN (KCA1_0626). An RpoN-dependent mannose PTS of *L. plantarum* WCFS1 with a similar operon structure to *L. pentosus* KCA1 has been characterized and can acts as a major regulator of carbohydrate uptake [49].

There are five complete putative two-component systems in KCA1, compared to four in *L. iners* AB-1. Four pairs have 100% amino acid identity to *L. pentosus* IG1 and *L. plantarum* strains, which includes histidine kinase *hpk1* (KCA1_0030) and response regulator *rrp1* (KCA1_0029). The fifth two-component response regulator TrxR (KCA1_2843), a transcriptional regulator of the AraC family without a corresponding histidine kinase, has beta-galactosidase (KCA1_2842) as its pair and appears to be horizontally acquired having a best BLASTp hit (58% id) to *Enterococcus casseliflavus* EC20.

### Transport

The *L. pentosus* KCA1 genome encodes over 100 putative genes for transport of cations, including three Nramp superfamily manganese transport proteins MntH (KCA1_1121, KCA1_2451, KCA1_0247) and a manganese ABC transporter MtsCBA (KCA1_0873–0875). Comparatively, *L. iners* AB-1 dedicates a large proportion of its genome [186 (15.6%) of protein-encoding genes] to transport [21]. In KCA1, a cluster of genes encodes an Fe-S assembly system including seven genes encoding three putative iron-sulfur assembly proteins SufB (KCA1_1250), SufD (KCA1_1247), and SufC (KCA1_1246). A novel DUF59 family Fe-S assembly SUF system protein, a putative aromatic ring hydroxylating enzyme involved in Fe-S cluster assembly (KCA1_1259), and a NifU family Fe-S cluster assembly scaffold protein SufE2 (KCA1_1249), were identified within the Fe-S loci.

There is also an iron chelatin ABC transporter (KCA1_1251–1253), iron ABC transporter (KCA1_1517–1519), a ferrichrome ABC transporter FhuGBCD (KCA1_2540–2543), and a ferrochelatase (KCA1_1122). It would be interesting to determine if *L. pentosus* KCA1 sequestration of iron limits availability of the metal to vaginal pathogens and enhances its ability to persist.

### Metabolism of Cofactors

The role of intestinal bacteria in the biosynthesis of vitamins and cofactors in the GIT was recognized as early as 1942 [50]. However, the contribution of vaginal lactobacilli to the biosynthesis of vitamins and cofactors, and their metabolic impact in the vagina has yet to be addressed. The genome of *L. pentosus* KCA1 dedicates 121 putative genes to metabolism of cofactors and vitamins including five genes for biotin biosynthesis. Twenty-four putative genes are involved in the biosynthesis of folate and eleven for pterines (molybdenum). A potential operon contains the riboflavin synthase alpha chain RibB (KCA1_1218), GTP cyclohydrolase II RibA (KCA1_1219) and 6,7-dimethyl-8-ribityllumazine synthase RibH (KCA1_1220), enzymes required for the first and last steps in the synthesis of riboflavin from GTP. Only one enzyme, the 5-amino-6-ribityl-aminouracil reductase, appears to be absent in *L. pentosus* KCA1 (**Figure S5**).

Like most lactobacilli, *L. pentosus* KCA1 appears to be incapable of complete *de novo* synthesis of pyridoxine (vitamin B6), as six genes are present including pyridoxal kinase (KCA1_0691), and phosphoserine aminotransferase SerC (KCA1_0179). All the enzymes required for the biosynthesis of coenzyme A from panthothenate are present in KCA1, as are those required for folate biosynthesis. The role of these cofactors/vitamins in the maintenance of vaginal health remains to be determined.

## Concluding Notes

The sequence of *L. pentosus* KCA-1 chromosome has revealed many interesting gene clusters or variants of known genes. It appears that the large ‘sugar life-style island’ has acquired gene cassettes for carbohydrate utilization from a variety of bacteria. In this island, there are many copies of genes encoding similar functions (transporters, enzymes) that appear not to be recent duplications, as they differ greatly in sequence and are most similar to ORFs in several different bacteria. The encoded putative functions suggest these gene cassettes may promote growth on a polyglycan substrate, potentially consisting of rhamnose, galacturonate, glucose, xylose, arabinose and glucuronate units. Novel putative genes identified include those for utilization of hydantoin, malonate, rhamnosides and utilization and assimilation of alkane-sulfonates. The *L. pentosus* KCA1 genome also encodes putative phage defense systems including CRISPRs and abortive infection, novel toxin-antitoxin systems, and biosynthesis of a novel antibacterial peptide, a class V cyclic bacteriocin precursor, here designated as pentocin KCA1 (*penA*). The genome provides a basis for future comparisons with *L. pentosus* strains from different ecological niches and women living in different geographic locations and against other vaginal *Lactobacillus* species.

## Materials and Methods

### Genome Sequencing and Assembly

Genomic DNA from *Lactobacillus pentosus* KCA1 was used to prepare a genomic library using the Illumina paired-end sample preparation protocol at the Centre for Applied Genomics, Toronto, Canada (www.tcag.ca). Paired-end sequencing was done with the Next-Generation Illumina GAII facility, utilizing an insert length of 450 bp. The 16,920,226 paired-end reads were assembled into contigs using the VELVET assembler tool (a detailed description of the organism, preparation, sequencing, DNA assembly and gap closure can be found in File S1 (*Supporting information Materials and Methods*). Mauve [51] and the Artemis Comparison Tool (ACT) [52] were used to evaluate the alignment and contig order between the *L. pentosus* KCA1, *L. pentosus* IG1 and *L. plantarum* WCFS1 genome data sets. The resulting 83 contigs (1 scaffold) were used for gene prediction with the help of GeneMark [53] and Glimmer software [54]. The protein-coding open-reading frames (ORFs) and RNA genes were functionally annotated using online automatic annotation pipelines including but not limited to RAST (Rapid Annotation using Subsystem Technology) [55], and subsequently manually curated using the Artemis and ACT tools [56], BLAST to the NCBI non-redundant data base, COG [22], LaCOG (Lactobacillales-specific Clusters of Orthologous protein coding Genes) [20] and metabolic predictions were made by KAAS (KEGG Automatic Annotation Server) [57] followed by manual improvement. The predicted ORFs were also submitted to Pfam [58] and TMHMM (http://www.cbs.dtu.dk/services/TMHMM/) for conserved domain and transmembrane domain predictions respectively. Predicted protein sequences from *L. pentosus* KCA1 were compared to the NCBI non-redundant database (nrdb) by BLAST_P_ for horizontal gene transfer. Genes were identified as foreign if the three most significant hits with E value less than or equal to 1.0×10^−20^ were a genus other than *Lactobacillus* with the most significant hit having at least 60% protein identity to the query sequence. For functional comparisons, the UniProt database (http://www.uniprot.org/BLASTp) was generally used with E-value cutoff of 1.0×10^−20^. For phylogeny, 16S rRNA sequences were aligned with MUSCLE (Multiple Sequence Comparison by Log-Expectation) [59], and unreliable positions were curated using Gblocks [60]. A maximum likelihood tree was generated by PhyML, which produced a log likelihood of −8926.84393 for 16S rRNA and a log likelihood of −11102.90190 for *recA,* using the GTR (General Time Reversible) nucleotide substitution model [61] and allowing 4 rate substitution categories. A Confidence value for the branching order was generated by bootstrapping (based on 100 replications).

The *L. pentosus* KCA1 whole genome shotgun (WGS) project has been deposited and released in the DNA Data Base in Japan (DDBJ)/European Molecular Biology Laboratory (EMBL)/GenBank under the accession AKAO00000000. The version described in this paper is the first version and consists of sequences AKAO01000001-AKAO01000083. (http://www.ncbi.nlm.nih.gov/bioproject/81575).

## Supporting Information

Figure S1 *COG distributions in the L. pentosus KCA1 genome.*
(TIFF)

Figure S2 *Comparative gene cassettes for utilization of hydantoines.*
(TIFF)

Figure S3 *Malonate utilization gene cassettes of L. pentosus KCA1 and other non-Lactobacillus bacteria.*
(TIFF)

Figure S4 *Comparison of genome organization surrounding the large cluster of EPS/CPS biosynthesis genes.* Genes are represented by arrows in forward and reverse strands. Shades of connecting bars indicate high sequence identity (bright red) to low sequence identity (pink). The blue connecting bars indicates a reverse orientation.
(TIFF)

Figure S5 *Metabolic pathway of riboflavin (vitamin B1) biosynthesis as predicted by KAAS.* The genes (EC numbers) for riboflavin are shaded in green.
(TIFF)

Table S1 **Ribosomal proteins encoded in L. pentosus KCA1 with the corresponding Codon Adaptation Index (CAI).**
(DOCX)

Table S2 **Sequence identity matrix/alignment for the housekeeping gene recA, pheS, dnaK, in selected L. plantarum and L. pentosus strains; recA alignment in selected Gram positive species.**
(DOCX)

Table S3 **Unique putative gene cassettes (relative to** ***L. plantarum*** **and** ***L. pentosus*** **IG1) for carbohydrate utilization predicted in** ***L. pentosus*** **KCA1.**
(DOCX)

Table S4 **Gene clusters for exopolysaccharide biosynthesis predicted in** ***L. pentosus*** **KCA1.**
(DOCX)

File S1 *Supporting information on materials and methods.*
(DOCX)
