# Supplementary figures and images for: Genome Sequence of Lactobacillus pentosus KCA1: Vaginal Isolate from a Healthy Premenopausal Woman
Source: PLoS One. 2013 Mar 19;8(3):e59239. doi: 10.1371/journal.pone.0059239 (PMC3602190; doi:10.1371/journal.pone.0059239)

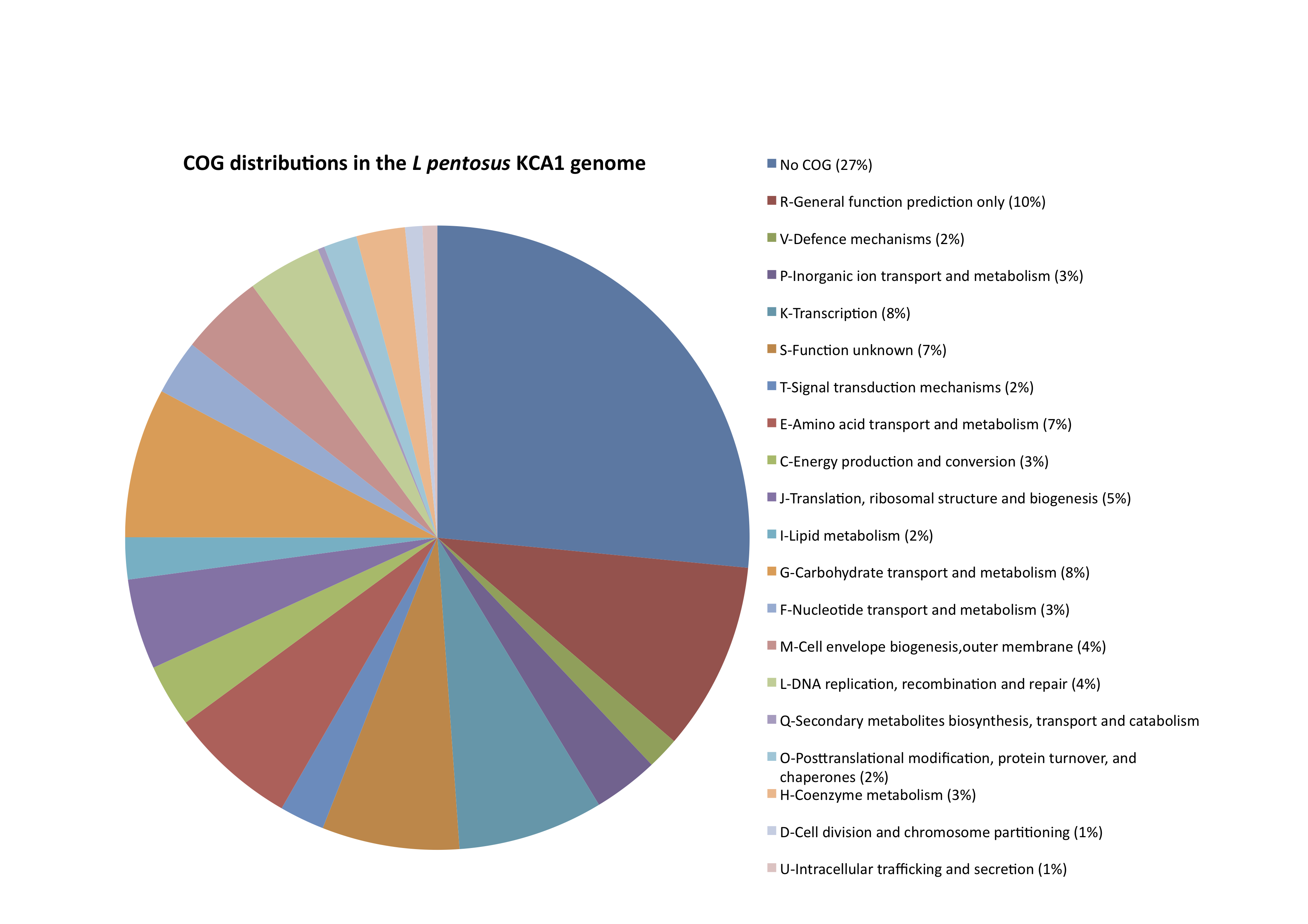

Supplement: Figure S1 — COG distributions in the L. pentosus KCA1 genome. (TIFF) [file pone.0059239.s001.tiff]

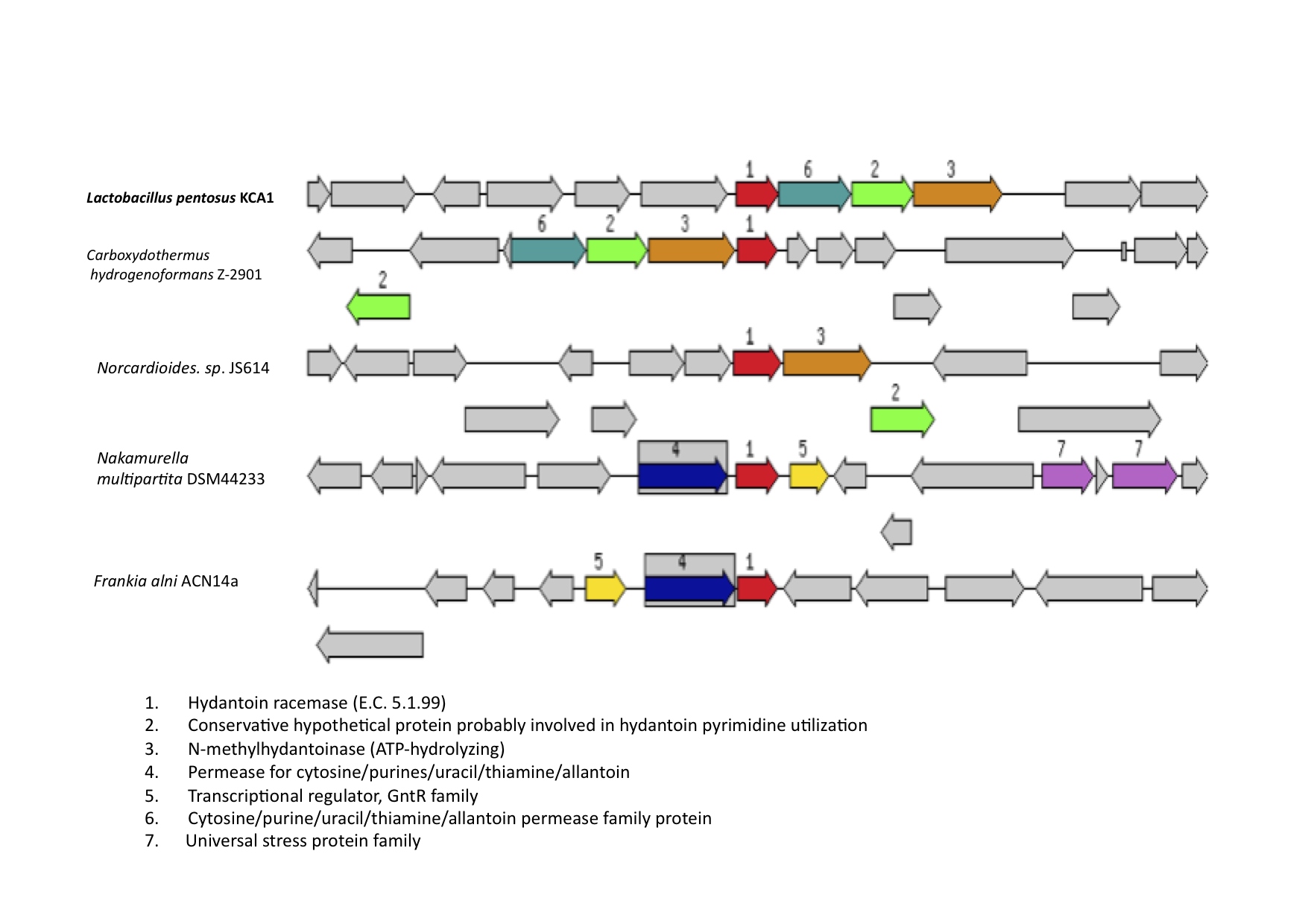

Supplement: Figure S2 — Comparative gene cassettes for utilization of hydantoines. (TIFF) [file pone.0059239.s002.tiff]

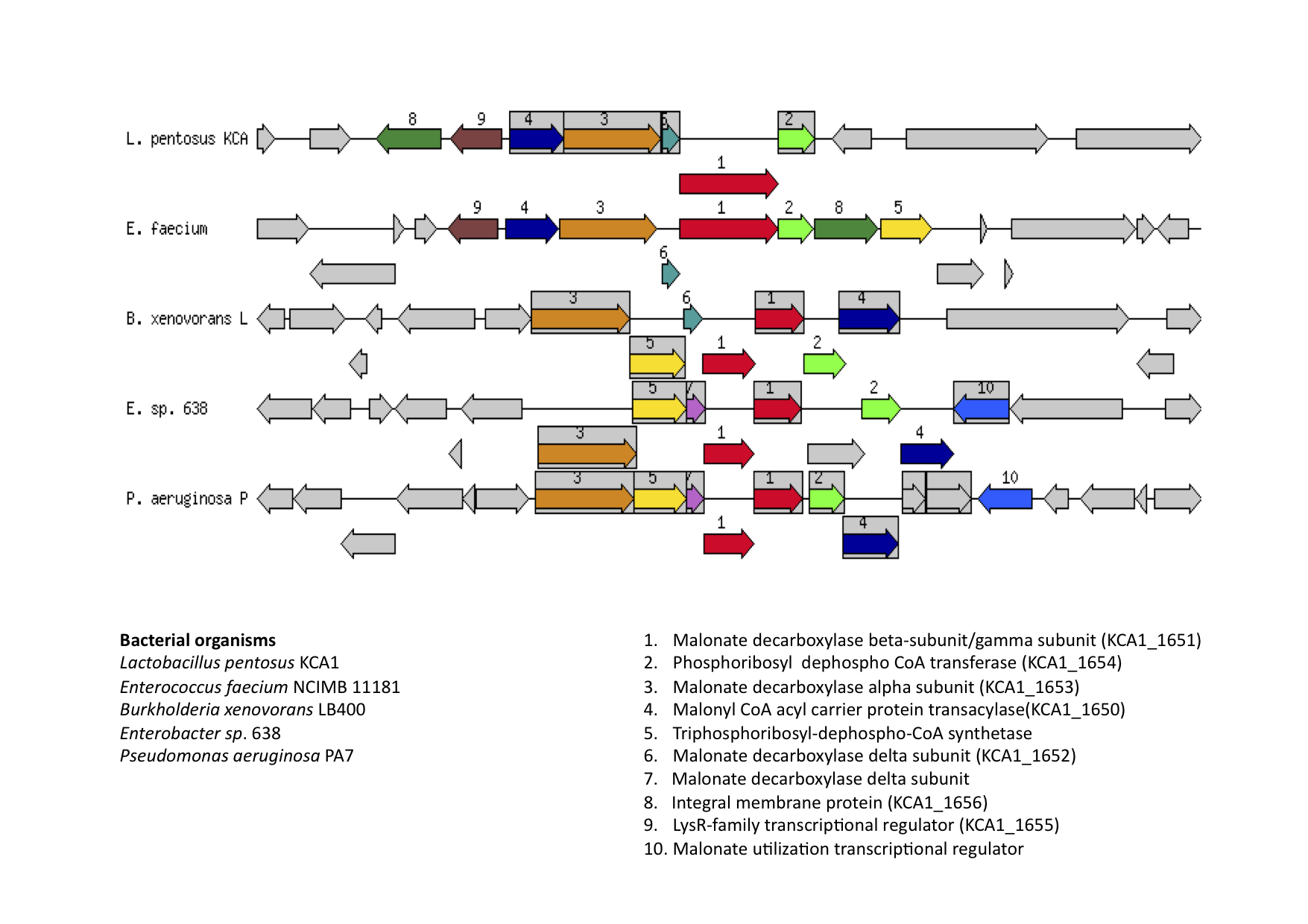

Supplement: Figure S3 — Malonate utilization gene cassettes of L. pentosus KCA1 and other non-Lactobacillus bacteria. (TIFF) [file pone.0059239.s003.tiff]

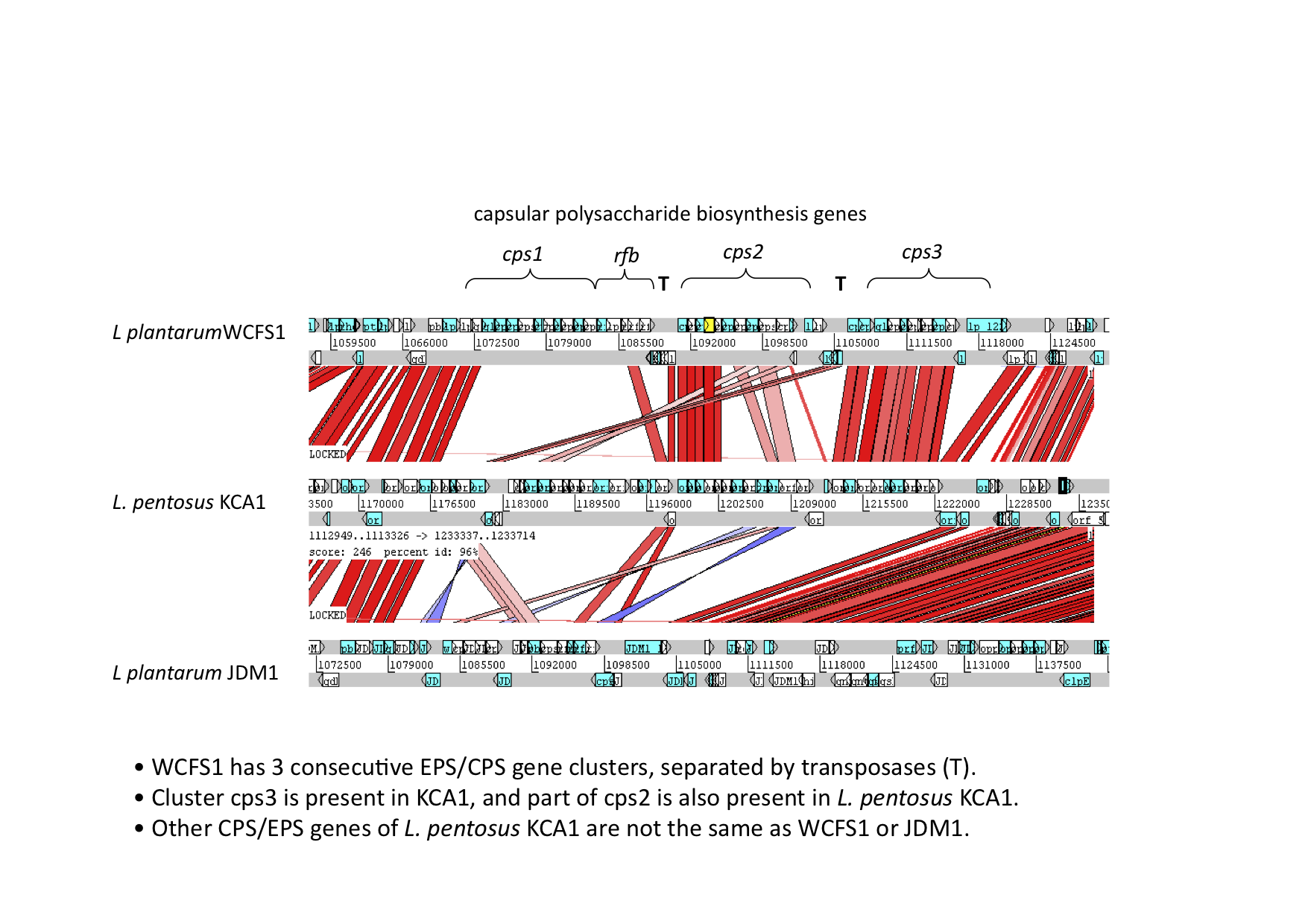

Supplement: Figure S4 — Comparison of genome organization surrounding the large cluster of EPS/CPS biosynthesis genes. Genes are represented by arrows in forward and reverse strands. Shades of connecting bars indicate high sequence identity (bright red) to low sequence identity (pink). The blue connecting bars indicates a reverse orientation. (TIFF) [file pone.0059239.s004.tiff]

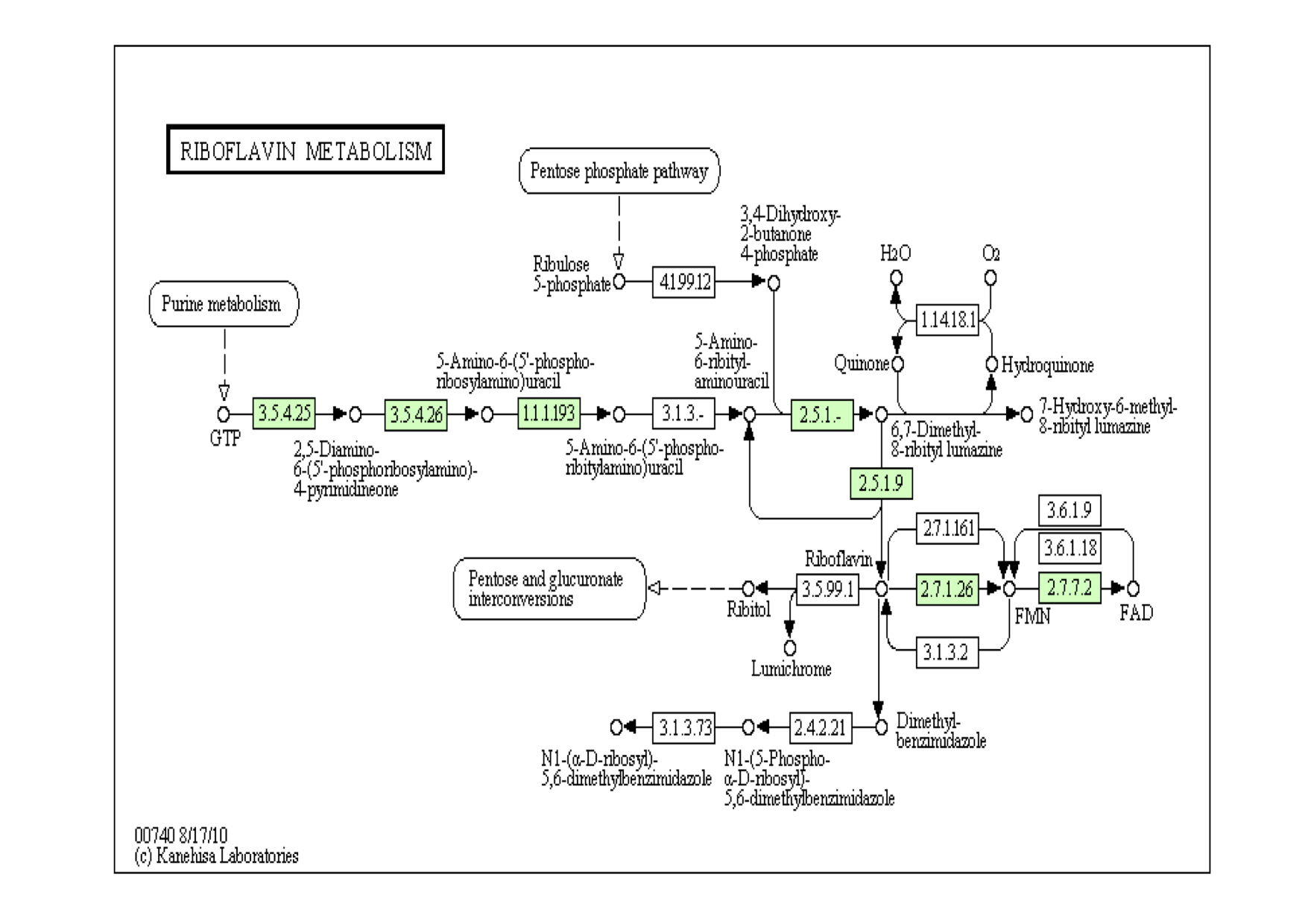

Supplement: Figure S5 — Metabolic pathway of riboflavin (vitamin B1) biosynthesis as predicted by KAAS. The genes (EC numbers) for riboflavin are shaded in green. (TIFF) [file pone.0059239.s005.tiff]
