# Supplementary material for: Genome Sequence of Lactobacillus pentosus KCA1: Vaginal Isolate from a Healthy Premenopausal Woman
Source: PLoS One. 2013 Mar 19;8(3):e59239. doi: 10.1371/journal.pone.0059239 (PMC3602190; doi:10.1371/journal.pone.0059239)
Supplement: Table S1 — Ribosomal proteins encoded in L. pentosus KCA1 with the corresponding Codon Adaptation Index (CAI). (DOCX) [file pone.0059239.s006.docx]

## Table S1: Ribosomal proteins encoded in *L. pentosus* KCA1 with the corresponding Codon Adaptation Index (CAI)

| **S/N** | **Ribosomal proteins encoded in *Lactobacillus pentosus* KCA1** | **CAI** |
| --- | --- | --- |
| 1 | Lactobacillus_pentosus_KCA1_0528 gnl\|CDD\|30430 COG0081, RplA, Ribosomal protein L1 | 0.727 |
| 2 | Lactobacillus_pentosus_KCA1_0821 gnl\|CDD\|30439 COG0090, RplB, Ribosomal protein L2 | 0.809 |
| 3 | Lactobacillus_pentosus_KCA1_0818 gnl\|CDD\|30436 COG0087, RplC, Ribosomal protein L3 | 0.698 |
| 4 | Lactobacillus_pentosus_KCA1_0819 gnl\|CDD\|30437 COG0088, RplD, Ribosomal protein L4 | 0.679 |
| 5 | Lactobacillus_pentosus_KCA1_0830 gnl\|CDD\|30443 COG0094, RplE, Ribosomal protein L5 | 0.792 |
| 6 | Lactobacillus_pentosus_KCA1_0833 gnl\|CDD\|30446 COG0097, RplF, Ribosomal protein L6P/L9E | 0.737 |
| 7 | Lactobacillus_pentosus_KCA1_0011 gnl\|CDD\|30708 COG0359, RplI, Ribosomal protein L9 | 0.647 |
| 8 | Lactobacillus_pentosus_KCA1_0529 gnl\|CDD\|30593 COG0244, RplJ, Ribosomal protein L10 | 0.79 |
| 9 | Lactobacillus_pentosus_KCA1_0527 gnl\|CDD\|30429 COG0080, RplK, Ribosomal protein L11 | 0.697 |
| 10 | Lactobacillus_pentosus_KCA1_1704 gnl\|CDD\|32445 COG2264, PrmA, Ribosomal protein L11 methylase | 0.697 |
| 11 | Lactobacillus_pentosus_KCA1_0530 gnl\|CDD\|30571 COG0222, RplL, Ribosomal protein L7/L12 | 0.853 |
| 12 | Lactobacillus_pentosus_KCA1_0856 gnl\|CDD\|30451 COG0102, RplM, Ribosomal protein L13 | 0.742 |
| 13 | Lactobacillus_pentosus_KCA1_0828 gnl\|CDD\|30442 COG0093, RplN, Ribosomal protein L14 | 0.725 |
| 14 | Lactobacillus_pentosus_KCA1_0837 gnl\|CDD\|30549 COG0200, RplO, Ribosomal protein L15 | 0.716 |
| 15 | Lactobacillus_pentosus_KCA1_0825 gnl\|CDD\|30546 COG0197, RplP, Ribosomal protein L16/L10E | 0.706 |
| 16 | Lactobacillus_pentosus_KCA1_0845 gnl\|CDD\|30552 COG0203, RplQ, Ribosomal protein L17 | 0.74 |
| 17 | Lactobacillus_pentosus_KCA1_0834 gnl\|CDD\|30605 COG0256, RplR, Ribosomal protein L18 | 0.702 |
| 18 | Lactobacillus_pentosus_KCA1_1404 gnl\|CDD\|30683 COG0335, RplS, Ribosomal protein L19 | 0.745 |
| 19 | Lactobacillus_pentosus_KCA1_1290 gnl\|CDD\|30640 COG0292, RplT, Ribosomal protein L20 | 0.735 |
| 20 | Lactobacillus_pentosus_KCA1_1357 gnl\|CDD\|30610 COG0261, RplU, Ribosomal protein L21 | 0.703 |
| 21 | Lactobacillus_pentosus_KCA1_0823 gnl\|CDD\|30440 COG0091, RplV, Ribosomal protein L22 | 0.655 |
| 22 | Lactobacillus_pentosus_KCA1_0820 gnl\|CDD\|30438 COG0089, RplW, Ribosomal protein L23 | 0.707 |
| 23 | Lactobacillus_pentosus_KCA1_0829 gnl\|CDD\|30547 COG0198, RplX, Ribosomal protein L24 | 0.801 |
| 24 | Lactobacillus_pentosus_KCA1_1359 gnl\|CDD\|30560 COG0211, RpmA, Ribosomal protein L27 | 0.708 |
| 25 | Lactobacillus_pentosus_KCA1_1389 gnl\|CDD\|30576 COG0227, RpmB, Ribosomal protein L28 | 0.694 |
| 26 | Lactobacillus_pentosus_KCA1_0826 gnl\|CDD\|30604 COG0255, RpmC, Ribosomal protein L29 | 0.714 |
| 27 | Lactobacillus_pentosus_KCA1_0836 gnl\|CDD\|32026 COG1841, RpmD, Ribosomal protein L30/L7E | 0.767 |
| 28 | Lactobacillus_pentosus_KCA1_1731 gnl\|CDD\|31549 COG1358, RPL8A, Ribosomal protein HS6-type (S12/L30/L7a) | 0.568 |
| 29 | Lactobacillus_pentosus_KCA1_0423 gnl\|CDD\|30603 COG0254, RpmE, Ribosomal protein L31 | 0.821 |
| 30 | Lactobacillus_pentosus_KCA1_0523 gnl\|CDD\|30616 COG0267, RpmG, Ribosomal protein L33 | 0.644 |
| 31 | Lactobacillus_pentosus_KCA1_1289 gnl\|CDD\|30639 COG0291, RpmI, Ribosomal protein L35 | 0.813 |
| 32 | Lactobacillus_pentosus_KCA1_1621 gnl\|CDD\|30885 COG0539, RpsA, Ribosomal protein S1 | 0.742 |
| 33 | Lactobacillus_pentosus_KCA1_1743 gnl\|CDD\|30401 COG0052, RpsB, Ribosomal protein S2 | 0.801 |
| 34 | Lactobacillus_pentosus_KCA1_0824 gnl\|CDD\|30441 COG0092, RpsC, Ribosomal protein S3 | 0.707 |
| 35 | Lactobacillus_pentosus_KCA1_1973 gnl\|CDD\|30868 COG0522, RpsD, Ribosomal protein S4 and related proteins | 0.806 |
| 36 | Lactobacillus_pentosus_KCA1_0835 gnl\|CDD\|30447 COG0098, RpsE, Ribosomal protein S5 | 0.733 |
| 37 | Lactobacillus_pentosus_KCA1_0007 gnl\|CDD\|30709 COG0360, RpsF, Ribosomal protein S6 | 0.826 |
| 38 | Lactobacillus_pentosus_KCA1_0815 gnl\|CDD\|30398 COG0049, RpsG, Ribosomal protein S7 | 0.759 |
| 39 | Lactobacillus_pentosus_KCA1_0832 gnl\|CDD\|30445 COG0096, RpsH, Ribosomal protein S8 | 0.791 |
| 40 | Lactobacillus_pentosus_KCA1_0857 gnl\|CDD\|30452 COG0103, RpsI, Ribosomal protein S9 | 0.726 |
| 41 | Lactobacillus_pentosus_KCA1_0817 gnl\|CDD\|30400 COG0051, RpsJ, Ribosomal protein S10 | 0.732 |
| 42 | Lactobacillus_pentosus_KCA1_0843 gnl\|CDD\|30449 COG0100, RpsK, Ribosomal protein S11 | 0.594 |
| 43 | Lactobacillus_pentosus_KCA1_0814 gnl\|CDD\|30397 COG0048, RpsL, Ribosomal protein S12 | 0.757 |
| 44 | Lactobacillus_pentosus_KCA1_0842 gnl\|CDD\|30448 COG0099, RpsM, Ribosomal protein S13 | 0.803 |
| 45 | Lactobacillus_pentosus_KCA1_1872 gnl\|CDD\|30548 COG0199, RpsN, Ribosomal protein S14 | 0.579 |
| 46 | Lactobacillus_pentosus_KCA1_1802 gnl\|CDD\|30533 COG0184, RpsO, Ribosomal protein S15P/S13E | 0.796 |
| 47 | Lactobacillus_pentosus_KCA1_1400 gnl\|CDD\|30577 COG0228, RpsP, Ribosomal protein S16 | 0.731 |
| 48 | Lactobacillus_pentosus_KCA1_0827 gnl\|CDD\|30535 COG0186, RpsQ, Ribosomal protein S17 | 0.756 |
| 49 | Lactobacillus_pentosus_KCA1_0009 gnl\|CDD\|30587 COG0238, RpsR, Ribosomal protein S18 | 0.698 |
| 50 | Lactobacillus_pentosus_KCA1_0822 gnl\|CDD\|30534 COG0185, RpsS, Ribosomal protein S19 | 0.739 |
| 51 | Lactobacillus_pentosus_KCA1_1803 gnl\|CDD\|30617 COG0268, RpsT, Ribosomal protein S20 | 0.697 |
| 52 | Lactobacillus_pentosus_KCA1_1688 30S ribosomal protein S21 (SSU ribosomal protein S21p) | 0.715 |
