# Supplementary material for: Genome Sequence of Lactobacillus pentosus KCA1: Vaginal Isolate from a Healthy Premenopausal Woman
Source: PLoS One. 2013 Mar 19;8(3):e59239. doi: 10.1371/journal.pone.0059239 (PMC3602190; doi:10.1371/journal.pone.0059239)
Supplement: Table S2 — Sequence identity matrix/alignment for the housekeeping gene recA, pheS, dnaK, in selected L. plantarum and L. pentosus strains; recA alignment in selected Gram positive species. (DOCX) [file pone.0059239.s007.docx]

## Table S2: Sequence identity matrix/alignment for the housekeeping gene recA, pheS, dnaK, in selected *L. plantarum* and *L. pentosus* strains; recA alignment in selected Gram positive species.

| **Sequence** | **plantarum type recA** | **plantarum WCFS1 recA** | **plantarum JDM1 recA** | **plantarum NC8 recA** | **arg type recA** | **pentosus type recA** | **pentosus IG1 recA** | **pentosus MP-10 recA** | **para type recA** | **pentosus KCA1 recA** |  |  |  |  |  |  |  |
| --- | --- | --- | --- | --- | --- | --- | --- | --- | --- | --- | --- | --- | --- | --- | --- | --- | --- |
| **plantarum type recA** | ID | 0.998 | 0.998 | 0.999 | 0.942 | 0.871 | 0.872 | 0.87 | 0.881 | 0.864 |  |  |  |  |  |  |  |
| **plantarum WCFS1 recA** | 0.998 | ID | 1 | 0.999 | 0.944 | 0.873 | 0.874 | 0.872 | 0.881 | 0.866 |  |  |  |  |  |  |  |
| **plantarum JDM1 recA** | 0.998 | 1 | ID | 0.999 | 0.944 | 0.873 | 0.874 | 0.872 | 0.881 | 0.866 |  |  |  |  |  |  |  |
| **plantarum NC8 recA** | 0.999 | 0.999 | 0.999 | ID | 0.943 | 0.872 | 0.873 | 0.871 | 0.882 | 0.865 |  |  |  |  |  |  |  |
| **arg type recA** | 0.942 | 0.944 | 0.944 | 0.943 | ID | 0.872 | 0.873 | 0.872 | 0.887 | 0.865 |  |  |  |  |  |  |  |
| **pent type recA** | 0.871 | 0.873 | 0.873 | 0.872 | 0.872 | ID | 0.999 | 0.999 | 0.859 | **0.968** |  |  |  |  |  |  |  |
| **pentosus IG1 recA** | 0.872 | 0.874 | 0.874 | 0.873 | 0.873 | 0.999 | ID | 0.998 | 0.86 | **0.969** |  |  |  |  |  |  |  |
| **pentosus MP-10 recA** | 0.87 | 0.872 | 0.872 | 0.871 | 0.872 | 0.999 | 0.998 | ID | 0.859 | **0.969** |  |  |  |  |  |  |  |
| **para type recA** | 0.881 | 0.881 | 0.881 | 0.882 | 0.887 | 0.859 | 0.86 | 0.859 | ID | 0.863 |  |  |  |  |  |  |  |
| **pentosus KCA1 recA** | 0.864 | 0.866 | 0.866 | 0.865 | 0.865 | **0.968** | **0.969** | **0.969** | 0.863 | ID |  |  |  |  |  |  |  |
|  |  |  |  |  |  |  |  |  |  |  |  |  |  |  |  |  |  |
| **Sequence** | **plantarum pheS** | **plantarum WCFS1 pheS** | **plantarum ST-III pheS** | **arg pheS** | **pentosus pheS** | **pentosus IG1 pheS** | **pentosus MP-10 pheS** | **para pheS** | **pentosus KCA1 pheS** |  |  |  |  |  |  |  |  |
| **plant pheS** | ID | 1,000 | 0.997 | 0.913 | 0.841 | 0.838 | 0.832 | 0.898 | 0.83 |  |  |  |  |  |  |  |  |
| **plantarum WCFS1 pheS** | 1,000 | ID | 0.997 | 0.913 | 0.841 | 0.838 | 0.832 | 0.898 | 0.83 |  |  |  |  |  |  |  |  |
| **plantarum ST-III pheS** | 0.997 | 0.997 | ID | 0.911 | 0.838 | 0.836 | 0.83 | 0.896 | 0.827 |  |  |  |  |  |  |  |  |
| **arg pheS** | 0.913 | 0.913 | 0.911 | ID | 0.832 | 0.83 | 0.83 | 0.876 | 0.834 |  |  |  |  |  |  |  |  |
| **pent pheS** | 0.841 | 0.841 | 0.838 | 0.832 | ID | 0.986 | 0.986 | 0.841 | **0.931** |  |  |  |  |  |  |  |  |
| **pentosus IG1 pheS** | 0.838 | 0.838 | 0.836 | 0.83 | 0.986 | ID | 0.986 | 0.838 | **0.929** |  |  |  |  |  |  |  |  |
| **pentosus MP-10 pheS** | 0.832 | 0.832 | 0.83 | 0.83 | 0.986 | 0.986 | ID | 0.832 | **0.931** |  |  |  |  |  |  |  |  |
| **para pheS** | 0.898 | 0.898 | 0.896 | 0.876 | 0.841 | 0.838 | 0.832 | ID | 0.838 |  |  |  |  |  |  |  |  |
| **pentosus KCA1 pheS** | 0.83 | 0.83 | 0.827 | 0.834 | **0.931** | **0.929** | **0.931** | 0.838 | ID |  |  |  |  |  |  |  |  |
|  |  |  |  |  |  |  |  |  |  |  |  |  |  |  |  |  |  |
| Sequence | plantarum 10069 dnaK | pentosus 11053 dnaK | pentosus 17972 dnaK | pentosus 17973 dnaK | **pentosus KCA1 dnaK** | pentosus MP-10 dnaK | pentosus IG1 dnaK |  |  |  |  |  |  |  |  |  |  |
| plantarum 10069 dnaK | ID | 0.892 | 0.892 | 0.89 | 0.898 | 0.893 | 0.893 |  |  |  |  |  |  |  |  |  |  |
| penosus t11053 dnaK | 0.892 | ID | 1,000 | 0.986 | 0.963 | 0.999 | 0.989 |  |  |  |  |  |  |  |  |  |  |
| pentosus 17972 dnaK | 0.892 | 1,000 | ID | 0.986 | 0.963 | 0.999 | 0.989 |  |  |  |  |  |  |  |  |  |  |
| pentosus 17973 dnaK | 0.89 | 0.986 | 0.986 | ID | 0.96 | 0.985 | 0.989 |  |  |  |  |  |  |  |  |  |  |
| **pentosus KCA1 dnaK** | 0.898 | **0.963** | **0.963** | **0.96** | ID | **0.963** | **0.962** |  |  |  |  |  |  |  |  |  |  |
| pentosus MP-10 dnaK | 0.893 | 0.999 | 0.999 | 0.985 | 0.963 | ID | 0.988 |  |  |  |  |  |  |  |  |  |  |
| pentosus IG1 dnaK | 0.893 | 0.989 | 0.989 | 0.989 | 0.962 | 0.988 | ID |  |  |  |  |  |  |  |  |  |  |
|  |  |  |  |  |  |  |  |  |  |  |  |  |  |  |  |  |  |
| **Sequences** | ***L. pentosus* KCA1** | ***L. plantarum* WCFS1** | ***L. pentosus* IG1** | ***L. pentosus* MP-10** | ***L. brevis* ATCC 367** | ***L.case*i ATCC 334** | ***L.delbrueckii* subsp.bulgaricus ATCC 365** | ***L. fermentum* IFO 3956** | ***L. gasseri* ATCC 33323** | ***L. helveticus* DPC 4571** | ***L.johnsonii* NCC 533** | ***L. rhamnosus* GG** | ***L. sakei* subsp.sakei 23K** | ***L. salivarius* UCC118** | ***L. lactis* subsp.lactis** | ***S. aureus* subsp.aureus** | ***B.subtilis* subsp. subtilis** |
| ***L.pentosus* KCA1** | ID | 80.299 | 95.95433 | 95.8663 | 68.7831 | 51.803 | 64.2342 | 65.5367 | 67.3973 | 65.3916 | 65.374 | 51.9789 | 72.2846 | 61.7414 | 54.7933 | 61.4943 | 62.7507 |
| ***L. plantarum* WCFS1** | 80.299 | ID | 80.2111 | 80.2111 | 68.1658 | 51.3561 | 63.7838 | 65.5367 | 64.3836 | 65.1184 | 64.4506 | 51.706 | 70.1311 | 60.28 | 55.818 | 61.2069 | 62.0821 |
| ***L. pentosus* IG1** | 95.95433 | 80.2111 | ID | 99.912 | 67.8131 | 51.4512 | 64.5946 | 65.6309 | 67.8539 | 66.9399 | 65.5586 | 51.9789 | 70.8801 | 62.6209 | 55.2331 | 62.2605 | 62.0821 |
| ***L. pentosus* MP-10** | 95.8663 | 80.2111 | 99.912 | ID | 67.8131 | 51.4512 | 64.6847 | 65.6309 | 67.3973 | 66.9399 | 65.4663 | 52.0668 | 70.7865 | 62.533 | 55.1451 | 62.1648 | 62.1777 |
| ***L. brevis* ATCC 367** | 68.7831 | 68.1658 | 67.8131 | 67.8131 | ID | 53.0864 | 63.4234 | 64.2185 | 64.8402 | 64.2987 | 62.0499 | 52.1164 | 69.6629 | 61.8166 | 58.5538 | 62.2605 | 61.2225 |
| ***L.casei* ATCC 334** | 51.803 | 51.3561 | 51.4512 | 51.4512 | 53.0864 | ID | 51.1712 | 54.049 | 52.3288 | 50.3643 | 51.5235 | 76.0101 | 55.3371 | 49.3892 | 51.0309 | 52.3946 | 53.4862 |
| ***L.delbrueckii* subsp.bulgaricus ATCC 365** | 64.2342 | 63.7838 | 64.5946 | 64.6847 | 63.4234 | 51.1712 | ID | 68.1733 | 66.0274 | 66.6667 | 64.4506 | 55.4054 | 64.9813 | 58.2883 | 52.7027 | 55.2682 | 57.4021 |
| ***L. fermentum* IFO 3956** | 65.5367 | 65.5367 | 65.6309 | 65.6309 | 64.2185 | 54.049 | 68.1733 | ID | 59.5104 | 58.7571 | 57.1563 | 51.9774 | 65.725 | 61.5819 | 53.7665 | 53.2567 | 58.5482 |
| ***L. gasseri* ATCC 33323** | 67.3973 | 64.3836 | 67.8539 | 67.3973 | 64.8402 | 52.3288 | 66.0274 | 59.5104 | ID | 75.9817 | 87.4423 | 50.5936 | 68.3521 | 66.21 | 58.9041 | 65.4215 | 60.2674 |
| ***L. helveticu*s DPC 4571** | 65.3916 | 65.1184 | 66.9399 | 66.9399 | 64.2987 | 50.3643 | 66.6667 | 58.7571 | 75.9817 | ID | 74.2382 | 53.3698 | 66.9476 | 65.3005 | 58.0146 | 63.5057 | 61.2225 |
| ***L.johnsonii* NCC 533** | 65.374 | 64.4506 | 65.5586 | 65.4663 | 62.0499 | 51.5235 | 64.4506 | 57.1563 | 87.4423 | 74.2382 | ID | 50.5078 | 66.4794 | 66.0203 | 58.6334 | 65.0383 | 60.8405 |
| ***L. rhamnosus* GG** | 51.9789 | 51.706 | 51.9789 | 52.0668 | 52.1164 | 76.0101 | 55.4054 | 51.9774 | 50.5936 | 53.3698 | 50.5078 | ID | 53.4644 | 49.2147 | 49.1409 | 50.4789 | 52.4355 |
| ***L. sakei* subsp.sakei 23K** | 72.2846 | 70.1311 | 70.8801 | 70.7865 | 69.6629 | 55.3371 | 64.9813 | 65.725 | 68.3521 | 66.9476 | 66.4794 | 53.4644 | ID | 68.4457 | 61.1423 | 62.4521 | 60.3629 |
| ***L. salivarius* UCC118** | 61.7414 | 60.28 | 62.6209 | 62.533 | 61.8166 | 49.3892 | 58.2883 | 61.5819 | 66.21 | 65.3005 | 66.0203 | 49.2147 | 68.4457 | ID | 58.6387 | 64.751 | 61.5091 |
| ***L. lactis* subsp.lactis** | 54.7933 | 55.818 | 55.2331 | 55.1451 | 58.5538 | 51.0309 | 52.7027 | 53.7665 | 58.9041 | 58.0146 | 58.6334 | 49.1409 | 61.1423 | 58.6387 | ID | 61.1111 | 58.3572 |
| ***S. aureus* subsp.aureus** | 61.4943 | 61.2069 | 62.2605 | 62.1648 | 62.2605 | 52.3946 | 55.2682 | 53.2567 | 65.4215 | 63.5057 | 65.0383 | 50.4789 | 62.4521 | 64.751 | 61.1111 | ID | 62.5479 |
| ***B.subtilis* subsp. subtilis** | 62.7507 | 62.0821 | 62.0821 | 62.1777 | 61.2225 | 53.4862 | 57.4021 | 58.5482 | 60.2674 | 61.2225 | 60.8405 | 52.4355 | 60.3629 | 61.5091 | 58.3572 | 62.5479 | ID |
