# Supplementary material for: Genome Sequence of Lactobacillus pentosus KCA1: Vaginal Isolate from a Healthy Premenopausal Woman
Source: PLoS One. 2013 Mar 19;8(3):e59239. doi: 10.1371/journal.pone.0059239 (PMC3602190; doi:10.1371/journal.pone.0059239)
Supplement: Table S3 — Unique putative gene cassettes (relative to L. plantarum and L. pentosus IG1) for carbohydrate utilization predicted in L. pentosus KCA1. (DOCX) [file pone.0059239.s008.docx]

## Table S3: Unique putative gene cassettes (relative to *L. plantarum* and *L. pentosus* IG1) for carbohydrate utilization predicted in *L. pentosus* KCA1

| **Gene cassettes for carbohydrate utilization** | |
| --- | --- |
| ***L. pentosus* KCA1 gene locus** | ***L. pentosus* KCA1 Product** |
| KCA1_2348-KCA1_2345 | Alfa-L-rhamnosidase |
|  | transport protein, major facilitator subfamily (MSF) |
|  | Beta-glucosidase , glycoside hydrolase family 3 |
|  | glycoside hydrolase family 43 |
|  | Transcriptional regulator, AraC family |
| KCA1_0727-KCA1_0725 | Transcriptional regulator, RpiR family |
|  | glucose/sucrose-specific PTS system, EIIBCA component |
|  | 6-phospho-beta-glucosidase |
| KCA1_2425-KCA1_2420 | transcriptional regulator, LacI family |
|  | hypothetical membrane protein |
|  | tannase (tannin acylhydrolase) |
|  | reverse transcriptase/maturase family protein |
|  | cell surface protein precursor, LPXTG-motif cell wall anchor |
|  | drug/metabolite transport protein, EamA family |
| KCA1_2562-KCA1_2567 | hypothetical protein |
|  | transcriptional regulator, GntR family |
|  | 6-phospho-beta-glucosidase |
|  | PTS system, cellobiose-specific IIC component |
|  | hypothetical protein |
|  | hypothetical protein |
| KCA1_2591-KCA1_2597 | Maltose O-acetyltransferase |
|  | Glucose uptake protein |
|  | Glucose 1-dehydrogenase |
|  | hypothetical protein |
|  | hypothetical protein |
|  | Permease, major facilitator superfamily |
|  | UDP-glucose 4-epimerase |
| KCA1_2847-KCA1_2844 | transcriptional regulator, AraC family |
|  | Beta-glucosidase-related glycosidase |
|  | Alfa-L-rhamnosidase |
|  | Beta-xylosidase |
| KCA1_2843-KCA1_2841 | transporter, major facilitator superfamily (MSF) |
|  | Beta-galactosidase |
|  | transcriptional regulator, AraC family |
