# Supplementary material for: Genome Sequence of Lactobacillus pentosus KCA1: Vaginal Isolate from a Healthy Premenopausal Woman
Source: PLoS One. 2013 Mar 19;8(3):e59239. doi: 10.1371/journal.pone.0059239 (PMC3602190; doi:10.1371/journal.pone.0059239)
Supplement: Table S4 — Gene clusters for exopolysaccharide biosynthesis predicted in L. pentosus KCA1. (DOCX) [file pone.0059239.s009.docx]

## Table S4: Gene clusters for exopolysaccharide biosynthesis predicted in *L. pentosus* KCA1.

| ***L. pentosus* KCA1 gene locus** | ***L. pentosus* KCA1 product** |
| --- | --- |
| KCA1_1223-KCA1_1224 | Cellulose synthase catalytic subunit [UDP-forming] |
|  | glycosyl transferase, family 2 |
| KCA1_0959-KCA1_0962 | hypothetical protein |
|  | Tyrosine-protein kinase |
|  | Beta-lactamase class C family protein |
|  | N-acetylmuramoyl-L-alanine amidase |
| KCA1_0963-KCA1_0965 | transposase, IS3 family |
|  | transposase, IS3 family |
|  | transposase, fragment |
| KCA1_0966-KCA1_0967 | Polysaccharide biosynthesis protein |
|  | Glycosyl transferase |
| KCA1_0968-KCA1_0972 | Glycerol-3-phosphate cytidylyltransferase |
|  | CDP-Glycerol:Poly(glycerophosphate) glycerophosphotransferase |
|  | capsular polysaccharide biosynthesis protein |
|  | polysaccharide polymerase |
|  | transferase, hexapeptide repeat |
| KCA1_0987-KCA1_0988 | Glycosyltransferase |
|  | polysaccharide biosynthesis protein |
| KCA1_0991-KCA1_0997 | Glycosyltransferase |
|  | polysaccharide biosynthesis protein, repeat unit transporter |
|  | hypothetical protein |
|  | N-acetylmuramoyl-L-alanine amidase |
|  | polysaccharide biosynthesis protein |
|  | glycosyltransferase, family 2 (GT2) |
|  | glycosyltransferase, family 2 (GT2) |
