# Supplementary material for: Genome Sequence of Lactobacillus pentosus KCA1: Vaginal Isolate from a Healthy Premenopausal Woman
Source: PLoS One. 2013 Mar 19;8(3):e59239. doi: 10.1371/journal.pone.0059239 (PMC3602190; doi:10.1371/journal.pone.0059239)
Supplement: File S1 — Supporting information on materials and methods. (DOCX) [file pone.0059239.s010.docx]

# Supporting information on Materials and Methods

## Organism

*Lactobacillus pentosus* KCA1 was originally isolated from the vagina of a healthy Nigerian woman. Initially, on the basis of a carbohydrate-fermentation test and information from 16S rRNA gene sequencing, this bacterium was identified as *Lactobacillus* *plantarum* KCA1 [1]. However, 16S ribosomal RNA sequences are not suitable for discrimination of *L. pentosus* and *L. plantarum* species because of the high identity value (99%) shared by *L. plantarum* and *L. pentosus* [2, 3]. Consequently, the definition of phylogenetic distances is also not feasible by such a classical approach for the *L. plantarum* group species. It has been proposed that the *recA* gene could be used as a phylogenetic marker [4, 5], and it has already given satisfying results for many bacterial genera. We re-classified the strain as *Lactobacillus pentosus* KCA1 on the basis of nucleotide sequences of the genes *recA* (recombinase A), *dnaK* (heat shock protein HSP70) and *pheS* (phenylalanyl-tRNA synthase alpha subunit) [6, 7, 8]. For *L. plantarum* and *L. pentosus*, phylogeny of these housekeeping genes has turned out to be the most useful marker for differentiation, and corroborates with other, more non-specific fingerprinting techniques such as RAPD and AFLP.

## Genomic DNA isolation and paired-end library preparation

The *L. pentosus* KCA1 strain was cultured in MRS agar (Sigma-Aldrich, Canada), and incubated at 37^o^C micro-aerophically for 24 hours. Preparation of *L. pentosus* KCA1 genomic DNA isolation was done using Epicentre MasterPure^TM^ DNA Purification Kit and dsDNA quality checked with an Eppendorf UV Biophotometer (California, USA). The genomic DNA libraries for the paired-end sequencing followed the Illumina protocol (Catalog #, PE-102-1001).

## Genome sequencing and assembly

Genomic DNA from *Lactobacillus pentosus* KCA1 was used to prepare a genomic library using the Illumina paired-end sample preparation protocol at the Centre for Applied Genomics, Toronto, Canada (www.tcag.ca). The sequencing was done with the Next-Generation Illumina GAII facility. The paired-end reads were filtered to incude only those with a Q score greater than 10 for all nucleotides, leaving 16,920,226 reads, about 8.45 million from each side with approximate insert size of 450 base-pairs used during sequencing. The paired-end reads were assembled into contigs with a maximum *kmer* length of 57 using the VELVET assembler tool. In the end, the final assembly has 602 nodes and n50 of 108429, a maximum contig size of 217,505 bp, and a total chromosome size of 3,418,159 bp. Thus almost all the reads were used for the assembly initially giving 281 contigs > 200bp in length, which were trimmed down to 83 contigs for gene predictions.

## Gene prediction and annotations

Open-reading frames (ORFs) greater than 100 nt were predicted using GeneMark [9] and Glimmer software [10]. The translated ORF predictions were compared to the NCBI non-redundant database (nrdb) using BLASTp to evaluate gene predictions. There were several cases of GeneMark predicted ORFs that were shorter in length compared to homologous sequences in the NCBI database, and the corresponding Glimmer prediction better matched the length of the proteins in the database. In these instances, the Glimmer prediction was preferentially retained over the GeneMark prediction in order to prevent overestimation of truncated pseudogenes. All predicted ORFs were manually checked with the Artemis software [11] and corrected when necessary (e.g. start codons, frameshifts).

The protein-coding ORFs and RNA genes were functionally annotated with the help of combined custom-created Perl scripts involving the online automatic annotation pipelines including but not limited to RAST (Rapid Annotation using Subsystem Technology) [12], BLAST to NCBI non-redundant data base, COG (Clusters of Orthologous Groups of proteins) [13], LaCOG (Lactobacillales-specific Clusters of Orthologous protein coding Genes) [14] and metabolic predictions were made by KAAS (KEGG Automatic Annotation Server) [15] followed by manual improvement. The predicted ORFs were also submitted to Pfam [16], InterProScan [www.ebi.ac.uk/Tools/pfa/iprscan] and TMHMM (http://www.cbs.dtu.dk/services/TMHMM/) for conserved domain and transmembrane domain predictions respectively. CRISPRs were analyzed with CRISPRFinder [17].

## Ordering of the contigs-scaffolds

Aligning the nucleotide sequences of *L. pentosus* KCA1 to *L. plantarum* WCFS1 with the tool Projector2 [http://bioinformatics.biol.rug.nl/websoftware/projector2/projector2] did not work very well, because the nucleotide sequence identity is rather variable and often below 85%, so that many contigs are not matched, even though the genomes are very co-linear. Therefore we decided to match on the protein level instead of nucleotide level to find the ordering of contigs. By matching at the protein level, most of the 84 contigs could be ordered according to the genome of *L. plantarum* WCFS1, and this included all the large scaffolds and contigs. The order of genes (synteny) is very similar over most of the genomes, despite the variable and low nucleotide sequence identity. There are only a few regions where rearrangements appear to occur relative to WCFS1. Mauve and ACT tools were used to evaluate the alignment and scaffold order between *L. pentosus* KCA1, *L. pentosus* IG1 and *L. plantarum* WCFS1 datasets [18, 11].

The read coverage was used to identify repeat regions. The read coverage for most of the contigs in the chromosome is about 200-fold (150-250 x coverage) and is fairly constant. All contigs that have a much higher coverage are probably repeats. The 5 rRNA operons in *L. plantarum* WCFS1 are essentially identical to *L pentosus* KCA1, and therefore these rRNA regions of *L. pentosus* KCA1 assembled into only 1 or 2 contigs, having a ~5x higher coverage. In this case we found the 16S rRNA in contig 27 (coverage 764x) and the 5S-23S rRNA in contig 15 (coverage 937x). Some tRNA regions with higher coverage are also found in rRNA gene clusters.

Contig 3 has an extremely high coverage (i.e. 2668x) and corresponds to a phage based on the encoded proteins, suggesting that it has 10x higher read coverage than the chromosome. This contig was be assembled in the ordered KCA1 chromosome at the corresponding position of one of the phages in the genome of strain WCFS1, but the 10x higher coverage suggests that it may not be present 10x in the chromosome (rather unlikely), but that it is also present as a separate phage genome.

In most cases the ordering of the *L. pentosus* KCA1 contigs and scaffolds is fairly certain, as their ordering corresponds exactly to the ordering of the genes of *L. plantarum* WCFS1. Primers were designed for the ends of each scaffold and long-range PCR (Expand Long Template PCR System, Roche) was used to verify connections between neighboring scaffolds as well as gaps within scaffolds. Eighty-seven PCR products representing the gaps were electrophoresed on a 1.5% agarose gel and bands were isolated, gel purified, and submitted for Sanger dideoxy chain termination sequencing (London Regional Genomics Institute, London, Ontario). Sanger reads were used to close some of the gaps.

## Prediction of highly expressed genes (using the codon adaptation index, CAI)

Very many microbial genomes reveal a codon usage bias or preference for a specific set of codons, in genes whose products are required in large quantities, which improves translation efficiency of these genes and contributes to optimizing cell growth [19, 20].

Using the EMBOSS [21] CAI tool, the ORF sequence of the small and large subunits of the ribosomal proteins were concatenated and used as a reference for calculating the codon adaptation index (CAI) of all the predicted genes in the *L. pentosus* KCA1 genome.

## Prediction of horizontal gene transfer (HGT)

Predicted protein sequences from *L. pentosus* KCA1 were compared to the NCBI nrdb by BLASTP. Excluding self-hits and hits to the same species, genes were identified as foreign if the three most significant hits (*E* <= 1E-20) were a genus other than *Lactobacillus* with the most significant hit having at least 60% protein identity to the query sequence.

## Comparative genomics

Comparative genomics of *L. pentosus* KCA1 was performed using genome sequences from the Genebank database for *L. pentosus* IG1, *L. pentosus* MP-10, and *L. plantarum* WCFS1, JDM1, ST-III, and ATCC 14917 respectively. Unfortunately, the ORF calling for *L. pentosus* IG1 is very poor, with hundreds of ORFs missed. Therefore, we manually improved the IG1 ORF calling before doing comparative genomics (data not shown). For functional comparisons, the UniProt database (<http://www.uniprot.org>/BLASTp) was generally used with E-value cutoff of 1.0x10^-20^. In addition, several individual sequences were imported into the Jalview [22] program for ClustalW and MAFFT alignment.

## Phylogenetic relationships to other *L. plantarum* and *L. pentosus* strains

The phylogenetic position of 15 Lactobacillus species and 4 Gram positive bacteria (*Bacillus subtilis*, *Staphylococcus aureus*, *Listeria monocytogenes* and *Lactococcus lactis*) was deciphered from 16S rRNA gene sequences obtained from the National Center for Biotechology Information (NCBI) database with the addition of the *L. pentosus* KCA1 16S rRNA sequence.

Sequences were aligned with MUSCLE (Multiple Sequence Comparison by Log Expectation) (23), and unreliable positions were curated using Gblocks (24). A maximum likelihood tree was generated by PhyML, which produced a log likelihood of -8926.84393, using the GTR (General Time Reversible) nucleotide substitution model (25) and allowing 4 rate substitution categories. Confidence values for the branching order were generated by bootstrapping (based on 100 replications).

## Prediction of cell-surface proteins (Secretome)

All predicted protein-coding sequences of *L. pentosus* KCA1 were imported into the online LAB-Secretome database (http://www.cmbi.ru.nl/ lab_secretome). The resultant cell-surface proteins present in *L. pentosus* KCA1 were automatically assigned indicating homologue species, e-values, subcellular localization, LaCOG classification and ORFans [26].

# References

1. Anukam KC, Osazuwa EO, Ahonkhai I, Reid G (2005). 16S rRNA gene sequence and phylogenetic tree of lactobacillus species from the vagina of healthy Nigerian women. African J Biotechnol. 4 (11): 1222-1227

2. Collins MD, Rodrigues UM, CAsh C, Aguirre M, Farrow JAE, (1991). Phylogenetic analysis of the genus Lactobacillus and related lactic acid bacteria as determined by reverse transcriptase sequencing of 16S rRNA. FEMS Microbiol Lett 77:5–12.

3. Quere F, Deschamps A, Urdaci MC (1997) DNA probe and PCR- specific reaction for *Lactobacillus plantarum*. J Appl Microbiol 82:783–790.

4. Eisen JA (1995). The RecA protein as a model molecule for the molecular systematic studies of bacteria: comparison of trees of RecAs and 16S RNA from the same species. J Mol Evol 41:1105–1123.

5. Lloyd AT, Sharp PM (1993) Evolution of the recA gene and the molecular phylogeny of bacteria. J Mol Evol 37:399–407.

6. Bringel F, Castioni A, Olukoya DK, Felis GE, Torriani S, Dellaglio F (2005) *Lactobacillus plantarum* *subsp. argentoratensis* *subsp. nov*., isolated from vegetable matrices. Int J Syst Evol Microbiol 55:1629-1634.

7. Huang CH, Lee FL, Liou JS (2010) Rapid discrimination and classification of the *Lactobacillus plantarum* group based on a partial *dnaK* sequence and DNA fingerprinting techniques. Antonie Leeuwenhoek 97(3): 289-296.

8. Naser SM, Dawyndt P, Hoste B, Gevers D, Vandemeulebroecke K, et al. (2007) Identification of lactobacilli by *pheS* and *rpoA* gene sequence analyses. Int J Syst Evol Microbiol 57(Pt 12): 2777-2789.

9. Isono K, McIninch JD, Borodovsky M (1994) Characteristic features of the nucleotide sequences of yeast mitochondrial ribosomal protein genes as analyzed by computer program GeneMark*.* DNA Res 1: 263- 269.

10. Salzberg S, Delcher A, Kasif S & White O (1998) Microbial gene identification using interpolated markov models*.* Nucl Acids Res 26: 544- 548.

11. Carver T, Berriman M, Tivey A, Patel C, Bohme U, et al. (2008) Artemis and ACT: viewing, annotating and comparing sequences stored in a relational database. Bioinformatics, 24(23):2672-2676

12. Aziz R*,* Bartels D, Best AA, DeJongh M, Disz T, *et al* (2008) The RAST server: Rapid annotations using subsystems technology*.* BMC Genomics 9: 75.

13.Tatusov R*,* Fedorova ND, Jackson JD, Jacobs AR, Kiryutin B, *et al* (2003) The COG database: An updated version includes eukaryotes*.* BMC Bioinformatics 4: 41.

14.Makarova K*,* Slesarev A, Wolf Y, Sorokin A, Mirkin B, *et al.* (2006) Comparative genomics of the lactic acid bacteria*.* Proc Natl Acad Sc USA 103: 15611- 15616.

15. Moriya Y, Itoh M, Okuda S, Yoshizawa AC & Kanehisa M (2007) KAAS: An automatic genome annotation and pathway reconstruction server*.* Nucl Acids Res 35: W182-185.

16. Finn RD*,* Mistry J, Tate J, Coggill P, Heger A, *et al* (2010) The pfam protein families database*.* Nucl Acids Res 38: D211-222.

17. Grissa L, Vergnaud G, Pourcel C (2007) CRISPRFinder: a web tool to identify clustered regularly interspaced short palindromic repeats. Nucl Acids Res 35: W52–W57.

18. Darling ACE, Mau B, Blattner FR & Perna NT (2004) Mauve: Multiple alignment of conserved genomic sequence with rearrangements*.* Genome Res 14: 1394-1403.

19. Sharp PM, Li W (1987) The codon adaptation index-a measure of directional synonymous codon usage bias, and its potential applications*.* Nucl Acids Res 15: 1281-1295.

20. Van Mandach C, Merkl R (2010) Genes optimized by evolution for accurate and fast translation encode in Archaea and Bacteria a broad and characteristic spectrum of protein functions. BMC Genomics 11:617.

21. Rice P, Longden I, Bleasby A (2000) EMBOSS: The European molecular biology open software suite*.* Trends in Genetics 16: 276-277.

22. Waterhouse AM, Procter JB, Martin DMA, Clamp M & Barton GJ (2009) Jalview version 2--a multiple sequence alignment editor and analysis workbench*.* Bioinformatics 25: 1189-1191.

23. Edgar RC (2004) MUSCLE: multiple sequence alignment with high accuracy and high throughput. Nucl. Acids Res 32 (5): 1792-1797.

24. Castresana J (2000) Selection of conserved blocks from multiple alignments for their use in phylogenetic analysis. Molecular Biol Evolution 17, 540-552

25. Guindon S, Dufayard JF, Lefort V, Anisimova M, Hordijk W et al (2010) New algorithms and methods to estimate maximum-likelihood phylogenies: assessing the performance of PhyML 3.0. Systematic Biol. 59(3):307-321.

26. Zhou M, Theunissen D, Wel M, Seizen RJ (2010). LAB-Secretome: a genome-scale comparative analysis of the predicted extracellular and surface- associated proteins of Lactic Acid Bacteria. BMC Genomics 11:651.
